# A pseudokinase version of the histidine kinase ChrS promotes high heme tolerance of *Corynebacterium glutamicum*

**DOI:** 10.3389/fmicb.2022.997448

**Published:** 2022-09-07

**Authors:** Aileen Krüger, Julia Frunzke

**Affiliations:** Forschungszentrum Jülich GmbH, Institute for Bio- and Geosciences 1, Jülich, Germany

**Keywords:** adaptive laboratory evolution (ALE), heme, pseudokinase, two-component system (TCS), histidine kinase, CA-domain

## Abstract

Heme is an essential cofactor for almost all living cells by acting as prosthetic group for various proteins or serving as alternative iron source. However, elevated levels are highly toxic for cells. Several corynebacterial species employ two paralogous, heme-responsive two-component systems (TCS), ChrSA and HrrSA, to cope with heme stress and to maintain intracellular heme homeostasis. Significant cross-talk at the level of phosphorylation between these systems was previously demonstrated. In this study, we have performed a laboratory evolution experiment to adapt *Corynebacterium glutamicum* to increasing heme levels. Isolated strains showed a highly increased tolerance to heme growing at concentrations of up to 100 μM. The strain featuring the highest heme tolerance harbored a frameshift mutation in the catalytical and ATPase-domain (CA-domain) of the *chrS* gene, converting it into a catalytically-inactive pseudokinase (ChrS_CA-fs). Reintroduction of the respective mutation in the parental *C. glutamicum* strain confirmed high heme tolerance and showed a drastic upregulation of *hrtBA* encoding a heme export system, conserved in Firmicutes and Actinobacteria. The strain encoding the ChrS pseudokinase variant showed significantly higher heme tolerance than a strain lacking *chrS.* Mutational analysis revealed that induction of *hrtBA* in the evolved strain is solely mediated *via* the cross-phosphorylation of the response regulator (RR) ChrA by the kinase HrrS and BACTH assays revealed the formation of heterodimers between HrrS and ChrS. Overall, our results emphasize an important role of the ChrS pseudokinase in high heme tolerance of the evolved *C. glutamicum* and demonstrate the promiscuity in heme-dependent signaling of the paralogous two-component systems facilitating fast adaptation to changing environmental conditions.

## Introduction

Heme constitutes 95% of functional iron in the human body and is a key molecule for almost all living cells (Ponka, 1999; Andrews et al., 2003) acting as cofactor for many important proteins, including cytochromes, hydroxylases, catalases, peroxidases (Ajioka et al., 2006; Layer et al., 2010), and serving as alternative iron source (Anzaldi and Skaar, 2010). Nevertheless, elevated levels of this iron-bound protoporphyrin are highly cytotoxic. While this toxicity partially originates from the redox-active iron, causing the formation of reactive oxygen species (Kumar and Bandyopadhyay, 2005), a not yet unraveled porphyrin-related toxicity is furthermore suggested (Stojiljkovic et al., 1999). Consequently, organisms have evolved sophisticated mechanisms ensuring heme homeostasis (Padmanaban et al., 1989; Anzaldi and Skaar, 2010). Among those, systems enhancing heme tolerance play an important role in both pathogenic and non-pathogenic prokaryotes. Known strategies include mechanisms of (i) heme sequestration (e.g., HemS of *Yersinia enteroliticia* Stojiljkovic and Hantke, 1994), (ii) heme degradation (e.g., IsdG of *Bacillus anthracis* Skaar et al., 2006), and (iii) heme export by the HrtBA system (Stauff and Skaar, 2009b; Heyer et al., 2012; Nakamura et al., 2022). The heme-dedicated ATP-binding cassette efflux pump HrtBA is a highly conserved system and predominantly found in Firmicutes and Actinobacteria (Krüger et al., 2022). Recent structural studies shed light on the mechanism HrtBA employs to sequester and extrude heme from the cytoplasmic membrane (Nakamura et al., 2022).

In Gram-positive bacteria, two-component systems (TCS) play a predominant role in the regulation of heme homeostasis (Bibb et al., 2007; Stauff and Skaar, 2009a; Hentschel et al., 2014; Keppel et al., 2019; Krüger et al., 2022). The prototypical TCS consists of a membrane-bound histidine kinase (HK), which undergoes autophosphorlyation at a conserved histidine residue upon stimulus perception. Subsequently, the phosphoryl group is transferred to a conserved aspartate residue of a cytoplasmic response regulator (RR) resulting in an appropriate output, e.g., altering gene expression (Stock et al., 2000; Mascher et al., 2006; Laub and Goulian, 2007). HKs may be composed of multiple domains with a significant architectural diversity, but typically consist of an N-terminal transmembrane domain and a C-terminal transmitter domain. The transmitter domain can be split up in the dimerization and histidine phosphotransfer (DHp) domain and the catalytical and ATPase (CA) domain (Dutta et al., 1999; Gao and Stock, 2009). The CA-domain comprises four sequence motifs, including N, G1, F, and G2 boxes, which bind ATP in a pocket using an ATP lid and are consequently necessary for the autophosphorylation reaction (Kim and Forst, 2001; Wolanin et al., 2002). The DHp-domain possesses the H box motif harboring the conserved histidine residue which is phosphorylated upon stimulus perception (Dutta et al., 1999), as well as the X box, which is required for dimerization. The DHp- and CA-domain are connected *via* a flexible linker, which probably also supports keeping the RR in place during the phosphotransfer reaction (Casino et al., 2009). Many HKs are bifunctional possessing also a phosphatase motif and subsequently acting both as kinase and phosphatase for the RR (Perego and Hoch, 1996; Laub and Goulian, 2007; Hentschel et al., 2014). Furthermore, also catalytically inactive variants of kinases have been identified, referred to as pseudokinases, that can act as important signaling modulators by various mechanisms (Raju and Shaw, 2015; Kung and Jura, 2019; Kwon et al., 2019; Mace and Murphy, 2021).

**FIGURE 1:**
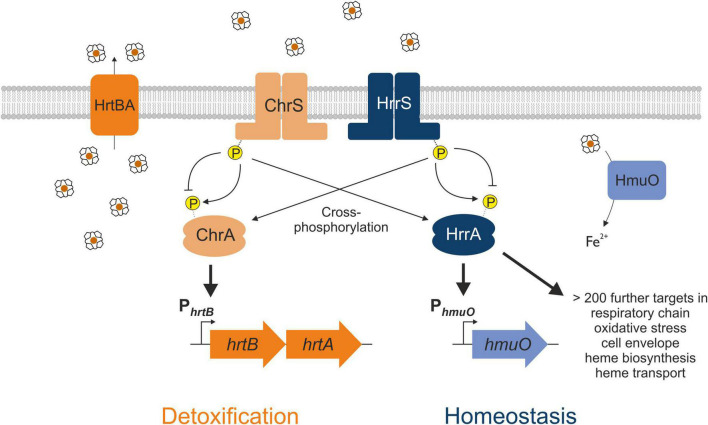
Interaction between the two heme-responsive two-component systems (TCS) ChrSA and HrrSA in *Corynebacterium glutamicum*. The simplified schematic representation shows the interaction of the two TCS ChrSA (orange) and HrrSA (blue) and each one representative target gene. ChrSA is solely responsible for the detoxification from heme *via* activating the *hrtBA* operon and autoregulates its own expression (Heyer et al., 2012). The HrrSA system was recently shown to act as regulator for heme homeostasis, controlling *hmuO* as well as more than 200 further genes involved in heme biosynthesis, respiratory chain, oxidative stress, or cell envelope remodeling (Keppel et al., 2020). The histidine kinases ChrS as well as HrrS undergo autophosphorylation in response to heme, activating the respective response regulator ChrA and HrrA. Cross-phosphorylation of the non-cognate response regulator has been demonstrated, while the phosphatase activity remains specific for the respective cognate response regulator (Hentschel et al., 2014).

Gene duplication events facilitate the evolution of TCS signaling enabling the integration of new input signals and diversification of the gene regulatory network. Members of the *Corynebacteriaceae* family, including the Gram-positive soil bacterium *Corynebacterium glutamicum*, represent an interesting example of a recent gene duplication event, encoding two paralogous two-component systems that both respond to the multifaceted molecule heme (Figure 1). After sensing heme availability *via* intramembrane interaction (Ito et al., 2009; Keppel et al., 2018), the TCS ChrSA acts as an activator of the *hrtBA* operon encoding the heme export system (Heyer et al., 2012). In contrast, the paralogous TCS HrrSA is a global regulator of heme homeostasis controlling more than 200 genomic targets including inter alia genes involved in heme biosynthesis, respiration as well as *hmuO*, encoding heme oxygenase (Keppel et al., 2020). Strikingly, a high level of cross-phosphorylation between the systems was observed (Keppel et al., 2019), while phosphatase activity of these HKs remains specific to their cognate RR (Hentschel et al., 2014).

In this study, we addressed the question how this underlying signaling cascade consisting of two paralogous TCSs facilitates fast adaptation to high heme levels, such as encountered by pathogenic species in the mammalian host, using the non-pathogenic *C. glutamicum* as a model. Understanding the mechanisms underlying microbial heme tolerance are not only important for the control of bacterial infections but also of biotechnological relevance for the engineering of a microbial production host demanding high product tolerance (Ko et al., 2021). Adaptive laboratory evolution (ALE) of *C. glutamicum* to increased heme levels resulted in the isolation of highly heme-tolerant clones bearing mutations in the CA-domain of ChrS. The catalytically inactive ChrS pseudokinase was shown to be required for the efficient activation of ChrA *via* the paralogous HK HrrS. Overall, this study demonstrated the potential of this ALE approach to provide new mechanistic insights in heme-dependent signaling and highlights the flexibility of paralogous TCS signaling facilitating the fast adaptation to enhanced heme levels.

## Materials and methods

### Bacterial strains and growth conditions

Bacterial strains used in this study are listed in Supplementary Table 1. For standard cultivation, *C. glutamicum* cells ATCC 13032 (wild type) and derivatives were streaked on agar plates (17 g/l) containing brain heart infusion (BHI) (Difco, BD, Heidelberg, Germany) (37 g/l) and inoculated at 30°C overnight. One single colony was picked and incubated for approximately 8 h at 30°C in 5 ml BHI in reaction tubes (for cultivation in shake flasks) or in 1 ml BHI in deep-well plates (VWR International, PA, United States) (for microtiter cultivation). This first pre-culture was used to inoculate the second pre-culture 1:10 in CGXII minimal medium (Keilhauer et al., 1993) supplemented with 2% (w/v) glucose but without any iron source to starve the cells from iron allowing the usage of heme as alternative iron source in the main culture. CGXII medium without FeSO_4_ is in the following referred to as “iron-free CGXII.” If appropriate, 25 μg/ml kanamycin was added to the medium. Incubation followed shaking at 120 rpm over night at 30°C. For the main experiment, cultures were inoculated to an OD_600_ of 1 in iron-free CGXII with 2% (w/v) glucose, and the respective amount of hemin (Sigma-Aldrich, St. Louis, United States). For simplicity, hemin is further referred to as heme throughout this study.

For the ALE experiment, the main culture was grown in deep-well plates for 1–3 days and then freshly transferred at an OD_600_ of 1 for the next batch. After the 13th inoculation, glycerol stocks of each population were frozen at −80°C. This allowed a restreaking of each potentially heterogeneous population on BHI-agar plates and picking of single evolved clones. Online monitoring of bacterial growth was performed using the BioLector^®^ microtiter cultivation system of Beckman Coulter GmbH (Baesweiler) (Kensy et al., 2009). Backscatter (a.u.) was measured in 30 min intervals as scattered light with a wavelength of 620 nm (gain: 20); YFP-fluorescence was measured at an excitation wavelength of 508 nm and an emission wavelength of 532 nm (gain: 80). Specific fluorescence (a.u.) was calculated by dividing the YFP-signal by the backscatter signal for each measurement.

*Escherichia coli* strains including DH5α and BTH101 were cultivated in Lysogeny Broth (10 g/l tryptone, 5 g/l yeast extract, 10 g/l NaCl) medium at 37°C in a rotary shaker and if needed for selection, 50 μg/ml kanamycin or 100 μg/ml ampicillin was added to the medium.

### Recombinant DNA work

Standard molecular methods were performed according to Sambrook and Russell (2001). For polymerase chain reactions (PCR) amplification of DNA fragments, chromosomal DNA of *C. glutamicum* ATCC 13032 was used as template and prepared as described previously (Eikmanns et al., 1994). Synthesis of oligonucleotides as well as sequencing was performed by Eurofins Genomics (Ebersberg, Germany).

Plasmids were constructed by amplifying DNA fragments using the respective oligonucleotides (Supplementary Tables 2, 3) and enzymatically ligated into a pre-cut vector backbone using Gibson assembly (Gibson et al., 2009).

For the deletion of genes in the genome of *C. glutamicum*, the suicide vector pK19-*mobsacB* was used (Schäfer et al., 1994). Electrocompetent *C. glutamicum* cells were transformed with the respective isolated plasmids by electroporation (van der Rest et al., 1999). Subsequently, the first and second recombination events were performed and verified as described in previous studies (Niebisch and Bott, 2001). The respective deletion was confirmed by amplification and sequencing.

### Whole genome sequencing

Whole genome resequencing of *C. glutamicum* strains isolated during the ALE experiment was performed using next generation sequencing (NGS). Genomic DNA was prepared using the NucleoSpin microbial DNA kit (Macherey-Nagel, Düren, Germany) according to manufacturer’s instructions. Concentrations of the purified genomic DNA were measured using Qubit 2.0 fluorometer (Invitrogen, Carlsbad, CA, United States) according to manufacturer’s instructions. The purified genomic DNA was used for the preparation for genome sequencing using NEBNext Ultra II DNA Library Kit for Illumina (New England BioLabs, Frankfurt am Main) and MiSeq Reagent Kit v2 (300-cycles) (Illumina, San Diego, CA, United States), according to manufacturer’s instructions. A MiSeq system (Illumina, San Diego, CA, United States) was used for sequencing. Data analysis and base calling were accomplished with the Illumina instrument software. FASTQ output files were analyzed for single nucleotide polymorphisms using PathoSystems Resource Integration Center (PATRIC) 3.6.12 (Davis et al., 2020).

### Gradient plates

For heme gradient plates, the different mutant strains were cultivated in triplicates as described above using deep-well plates for the first and second pre-culture. Subsequently, cultures were harvested and resuspended to an OD_600_ of 1 in 0.9% NaCl. For each spot, 2 μl of the respective samples were spotted on the gradient plates. The gradient plates were always prepared freshly. Therefore, 30 ml of iron-free CGXII with 2% (w/v) glucose, and 17 g/l Bacto Agar (Difco, BD, Heidelberg, Germany) was poured into a squared agar plate, which was in inclined position. Then, after drying of the first layer, the incline was removed and further 30 ml of iron-free CGXII containing 2% glucose, and 15 μM heme were poured in the plates so that a heme gradient results.

### Bacterial two-hybrid assays

#### Bacterial two-hybrid plate assays for the qualitative assessment of protein-protein interactions

Bacterial two-hybrid assays were performed based on the BACTH kit according to manufacturer’s instructions (Euromedex, Souffelweyersheim, France). This method is based on the two fragments T25 and T18 of the catalytical domain of the adenylate cyclase from *Bordetella pertussis*, which is only active when these two fragments are physically in close contact. Therefore, T25 and T18 were each fused once to ChrS, ChrS-Ala245fs, and HrrS. If the HKs interact with each other, this allows a functional complementation of T25 and T18, leading to cAMP synthesis, which binds to the catabolic activator protein (CAP). cAMP/CAP complexes are pleiotropic regulators of gene transcription in *E. coli* and therefore turn on the expression of e.g., the *lac* operon.

Therefore, *E. coli* BTH101, which lack adenylate cyclase activity, were transformed with two plasmids of heterologous proteins fused once to T25 and once to T18. This approach was directly diluted and spotted as 10^0^, 10^–1^, and 10^–2^ dilutions on LB plates containing 40 μg/ml X-Gal, 50 μg/ml kanamycin, 100 μg/ml ampicillin, and 0.5 mM IPTG and incubated approximately 24 h at 30°C. Bacteria producing interacting proteins will form blue colonies. Otherwise, the colonies remain white. This allowed to check also for heterogeneity in expression. Additionally, the approach was also plated on LB plates only with antibiotics, to allow picking for further biological replicates for the β-galactosidase assay.

#### Bacterial two-hybrid β-galactosidase measurements

Transformed *E. coli* strains (compare 1.5.1) were re-cultivated in deep-well plates as triplicates in LB media with 50 μg/ml kanamycin, 100 μg/ml ampicillin and 0.5 mM IPTG. OD_600_ of the overnight cultures was measured in a Tecan Reader (Thermo Fisher Scientific, Massachusetts, United States). The β-galactosidase assay was adapted according to a previous study for 96-well plates (Griffith and Wolf, 2002). Per sample, 1 ml Z-buffer (60 mM Na_2_HPO_4_ × 12H_2_O, 40 mM NaH_2_PO_4_ × H_2_O, 10 mM KCl, 1 mM MgSO_4_ × 7H_2_O, 50 mM β-mercaptoethanol) was mixed with 20 μl 0.1% SDS, 40 μl chloroform and 200 μl of cell culture. The solution was resuspended 15 times for permeabilization. 100 μl of the permeabilized cells were put into a microtiter plate, where each 20 μl of 4 mg/ml o-nitrophenol-ß-galactosidase (ONPG) was added. When a yellowish color was observable (10 min), 50 μl of 1 M Na_2_CO_3_ was added for reaction termination. Using the Tecan Reader (Thermo Fisher Scientific, Massachusetts, United States), A_420_ and A_550_ was measured. The Miller Unit, representing the standardized amount of β-Gal activity, was calculated using the following formula:


(1) $$1\,M\,U=1000*\frac{\left(A_{420}-\left(A_{550}*1.75\right)\right)}{\left(t*v*A_{600}\right)}$$

With A_420_ being the absorbance of the yellow o-nitrophenol, A_550_ the scatter from the cell debris, 1.75 is the factor which needs to be multiplied with A_550_ to approximate scatter observed at 420 nm, t is time in minutes, v is the volume of the culture employed in the plate and A_600_ for the cell density. The value of A_600_ was calibrated to proper OD_600_.

### DNA microarrays

For the analysis of the transcriptome, *C. glutamicum* wild type and *C. glutamicum* ChrS-Ala245fs were cultivated in triplicates as described above in 50 ml CGXII medium containing 2% glucose, no FeSO_4_ and 4 μM heme in shake flasks. Cells were harvested after 6 h, when the wild type reached an OD_600_ around 2.5 and the mutated strain around 5. The cell suspension was centrifuged at 4,250 × g, 10 min, 4°C in falcons filled with ice. The resulting pellets were frozen in liquid nitrogen and stored at −80°C. The following RNA preparation, cDNA synthesis, microchip hybridization, scanning, and overall evaluation was performed as described in previous studies (Baumgart et al., 2013). The microarray data described in this study are available at NCBI’s Gene Expression Omnibus under the GEO accession number GSE206796.

## Results

### Adaptive laboratory evolution of *Corynebacterium glutamicum* toward tolerance of high heme levels depends on the heme exporter HrtBA

**FIGURE 2:**
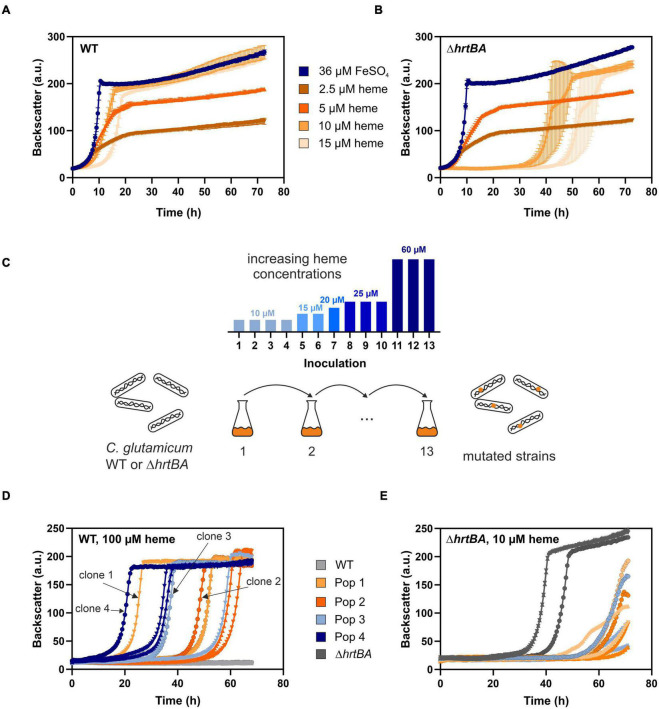
Adaptive laboratory evolution of *C. glutamicum* to high heme levels. The *C. glutamicum* wild type (WT) **(A)** as well as the Δ*hrtBA* strain **(B)** were inoculated at a starting-OD_600_ of 1 in CGXII medium containing 2% glucose and either 36 μM iron (blue) or increasing concentrations of heme (2.5–15 μM, shades of orange). Data represent the average of three biological replicates including standard deviations depicted as error bars. **(C)** Schematic representation of the adaptive laboratory evolution (ALE) experiment. The heme concentration was increased from 10 to 60 μM heme for the WT in overall 13 repetitive batch cultures (depicted in the bar graph). For the Δ*hrtBA* strain, concentrations > 15 μM remained toxic throughout the experiment and therefore were not further increased. **(D)** Growth of each three single clones derived from four different evolved *C. glutamicum* wild type populations on 100 μM heme. **(E)** Growth of each three single clones of the three evolved Δ*hrtBA* populations on 10 μM heme.

Previous studies reported a crucial role of the two-component system ChrSA for heme tolerance of *C. glutamicum* (Heyer et al., 2012). Figures 2A,B show characteristic growth curves of the *C. glutamicum* wild type (Figure 2A) and the deletion mutant Δ*hrtBA* (Figure 2B) on increasing concentrations of heme. Growing on standard conditions with 36 μM FeSO_4_ as iron source, the wild type displayed a growth rate of 0.42 ± 0.01 h^–1^. Lower heme concentrations between 2.5 and 5 μM showed strongly reduced growth rates of 0.16 ± 0.003 and 0.20 ± 0.003 h^–1^, respectively, and reduced backscatter levels compared to the cultivation under non-limiting conditions. Higher concentrations between 10 and 15 μM heme exhibited restored backscatter levels and growth rates of 0.22 ± 0.005 and 0.27 ± 0.012 h^–1^, but were accompanied by an elongated lag phase as a result of heme toxicity. This effect of toxicity was even more evident for the growth of a strain lacking the operon *hrtBA*. While growth on standard conditions and low concentrations of heme were comparable to the WT, higher concentrations led to a significantly elongated lag phase of ∼35 and 50 h, respectively.

To elucidate mechanisms promoting high heme tolerance, we performed an ALE experiment applying increasing concentrations of heme to *C. glutamicum* wild type and the Δ*hrtBA* strain (Figure 2C). The ALE was accomplished in CGXII minimal medium, with 13 repetitive batch cultivations and started from each four independent single colonies, yielding four evolving populations. Batch cultures were started on 10 μM heme and were stepwise increased to finally reach 60 μM heme in the case of *C. glutamicum* wild type. From *C. glutamicum* wild type populations, four single clones were isolated from agar plates after the 13th batch cultivation and then further characterized in liquid culture (Figure 2D). Further analysis on earlier inoculation steps was also performed (Supplementary Figure 1). Remarkably, all isolates from the 13th inoculation were able to grow in the re-cultivation on medium containing up to 100 μM heme where growth of the parental strain was completely inhibited. By contrast, heme levels above 15 μM heme remained toxic to the Δ*hrtBA* strain and could not be increased throughout the ALE experiment without killing the cells. Only three of the four starting populations survived 13 inoculations and for these, no significant adaptation was observed throughout the ALE experiment (Figure 2E). These results already underlined that HrtBA represents a key factor for the adaptation of *C. glutamicum* to high heme levels.

### Mutations in the catalytic- and ATPase domain of ChrS lead to significantly improved growth on heme

Whole genome sequencing of the four isolated clones from the ALE experiment revealed that all of them possess a mutation in the gene encoding the HK ChrS (Table 1). Remarkably, in all four cases, the catalytic- and ATPase (CA) domain was affected. For clone 2, a stop codon was inserted directly at the beginning of the CA-domain, while clone 3 possesses an amino acid exchange inside and close to the start of this domain. Strikingly, clone 1 and 4 (evolved in two independent cell lines) showed the exact same frameshift mutation for alanine at position 245, immediately after the dimerization and histidine phosphotransfer (DHp) domain of ChrS. This mutation also affects the linker between the CA- and DHp-domain. Since the strains carrying this mutation showed the highest heme tolerance, evolved clone number 1, in the following referred to as 1.fs, was further analyzed within this study.

**TABLE 1:** Key mutations identified in *C. glutamicum* strains featuring increased heme tolerance.

Clone	Type	Variance nucleotides	Variance amino acids	Locus
1.fs, 4.fs	Frameshift	733delG	Ala245fs	cg2201 Sensor histidine kinase **ChrS**
2.*	Non-sense	862C > T	Gln288*	
3. <	Missense	916A > C	Thr306Pro	

The * is the symbol for the insertion of a stop-codon. Similar, the > indicates an amino acid exchange.

**FIGURE 3:**
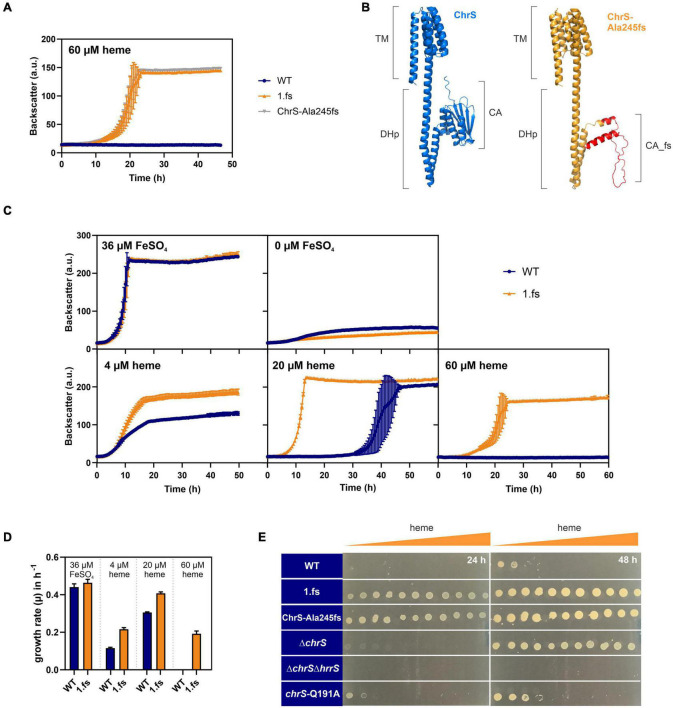
The ChrS-Ala245fs pseudokinase promotes heme tolerance. Data represent the average of three biological replicates including standard deviations depicted as error bars. Cells were inoculated at a starting-OD_600_ of 1 in CGXII medium containing 2% glucose and the indicated amount of heme or iron. **(A)** Growth of *C. glutamicum* carrying the reintegrated ChrS-Ala245fs allele (gray) compared to the evolved clone 1.fs (orange) and the wild type (WT) (blue). **(B)** Predicted protein structures of ChrS (blue) and the truncated ChrS-Ala245fs variant (orange, with 60 additional amino acids caused by the frameshift, shown in red). Prediction was performed using AlphaFold2 (Jumper et al., 2021; Varadi et al., 2021). Domain arrangements are shown next to the protein structures. TM, transmembrane domain; DHp, dimerization- and histidine-phosphotransfer-domain; CA, catalytic- and ATPase-domain. **(C)** Growth of the WT (blue) and the evolved clone 1.fs (orange) on different heme and iron concentrations; further conditions shown in Supplementary Figure 2. **(D)** Comparison of growth rates μ in h^– 1^ of WT (blue) and 1.fs (orange) at different heme and iron conditions. **(E)** Different strains were spotted on heme gradient plates. The WT was compared to the evolved clone (1.fs), the reintegration for the evolved clone (ChrS-Ala245fs), the *chrS* deletion strain (Δ*chrS*), the *chrS* and *hrrS* deletion strain (Δ*chrS* Δ*hrrS*) and the phosphatase mutant (*chrS*-Q191A). Photos of plates were taken after 24 and 48 h. A representative experiment out of three is shown.

Reintegration of this mutation into the wild type parental strain confirmed that this frameshift mutation in *chrS* led to highly improved growth on heme (Figure 3A and Supplementary Figure 2). Protein structure prediction *via* AlphaFold2 shows the truncated CA-domain of ChrS-Ala245fs with additional 60 amino acids caused by the frameshift resulting in a presumably catalytically-inactive pseudokinase variant (Figure 3B; Jumper et al., 2021; Varadi et al., 2021).

The 1.fs strain showed normal growth under standard conditions. However, it outcompeted the wild type in the presence of heme at all concentrations tested, but not under iron starved conditions (Figures 3C,D and Supplementary Figure 2). A heme gradient plate experiment further confirmed the improved heme tolerance of the evolved clone 1.fs, and showed that this is also the case for the reintegrated pseudokinase variant ChrS-Ala245fs (Figure 3E).

### The ChrS pseudokinase promotes increased *hrtBA* expression and is crucial for the improved growth on heme

**FIGURE 4:**
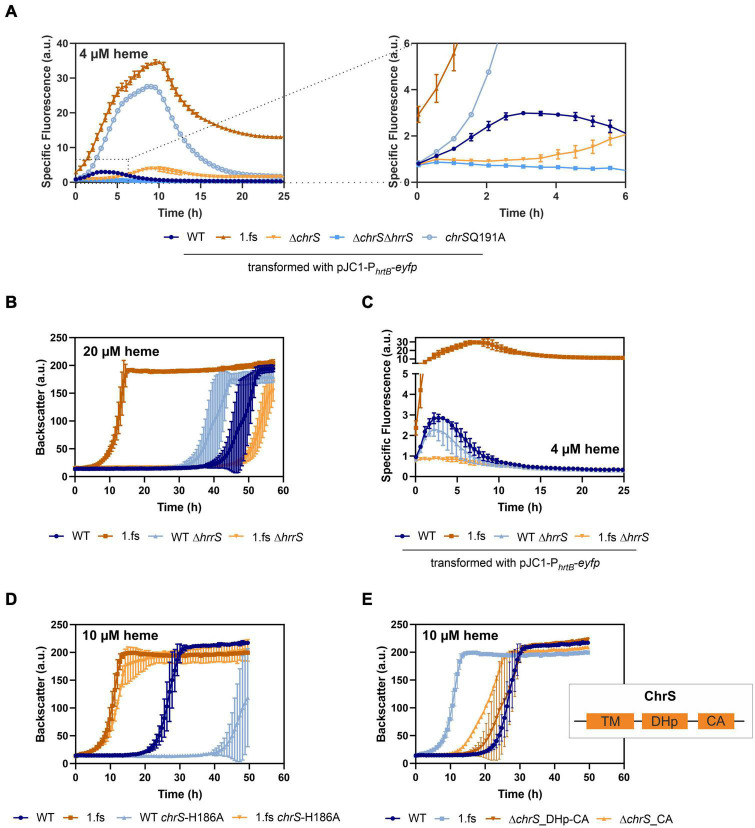
Mutational analysis provides insights in the *hrtBA* activating cascade of the evolved *C. glutamicum* 1.fs clone. Data represent the average of three biological replicates including standard deviations depicted as error bars. Cells were inoculated at a starting-OD_600_ of 1 in CGXII medium containing 2% glucose and the indicated amount of heme or iron. **(A)** Reporter assays visualizing *hrtBA*-expression using the plasmid pJC1-P*_*hrtB*_*-*eyfp* for transformation of the wild type (WT) (dark blue), the evolved clone 1.fs (dark orange), Δ*chrS* (light orange), Δ*chrS*Δ*hrrS* (mid-blue), and *chrS*-Q191A (light blue). **(B)** Growth of the evolved clone (shades of orange) and the WT (shades of blue) natively or with each a deletion of *hrrS*. **(C)** Reporter assays for *hrtB*-expression using pJC1-P*_*hrtB*_*-*eyfp* upon deletion of *hrrS* in 1.fs (shades of orange) and the WT (shades of blue). **(D)** Growth of the wild type strain (shades of blue) and the evolved 1.fs strain (shades of orange) natively or possessing an amino acid exchange of the autophosphorylation histidine (H^186^) of ChrS. **(E)** Impact of ChrS truncation on growth; ChrS variants lacking only the CA-domain (light orange) or CA-domain and DHp-domain (dark orange) compared to the WT and 1.fs strain (shades of blue). TM, transmembrane domain; DHp, dimerization- and histidine-phosphotransfer-domain; CA, catalytic- and ATPase-domain.

The main known target of ChrA is the *hrtBA* operon encoding a heme export system. To unravel the impact of the frame-shift mutation in *chrS* on its activation kinetics, *hrtBA* reporter assays were performed, using the reporter plasmid pJC1-P*_*hrtBA*_*-*eyfp* (Heyer et al., 2012). In fact, reporter assays (Figure 4A) showed a > 10-fold elevated expression of *hrtB* in the evolved clone 1.fs compared to the WT. This also applies for strain carrying the reintegrated gene variant (Supplementary Figure 3A) and was further confirmed by qPCR (Supplementary Figure 4). Strikingly, *hrtBA* expression was constitutively high also during cultivation under standard conditions, i.e., without external addition of heme (Supplementary Figure 3).

In the following, we further analyzed the growth and *hrtBA* expression of the evolved clone in comparison to different mutant strains, including the deletion mutants Δ*chrS* and Δ*chrS*Δ*hrrS* as well as the phosphatase mutant *chrS*-Q191A (Figures 3E, 4A; Hentschel et al., 2014). Heme gradient plates showed that the complete deletion of the *chrS* gene also led to an improved growth compared to the WT. This is caused by the abolished dephosphorylation of ChrA by its cognate kinase/phosphatase ChrS, allowing constant *hrtBA* expression due to cross talk with HrrS. The delay in *hrtB* expression is explained by the less sensitive response of HrrS to heme (Keppel et al., 2019). Interestingly, the Δ*chrS* deletion strain grew worse than the evolved strain 1.fs. This is in line with the lower *hrtBA* expression level in comparison to 1.fs. These results indicated that the remaining part of ChrS, which is present in the evolved clone, must play a crucial role for this high activation. Strikingly, 1.fs also significantly outperformed the phosphatase mutant *chrS*-Q191A in terms of growth and *hrtBA* expression.

Considering that the frame-shift mutation in *chrS* likely abolished the catalytic activity of the ChrS kinase, we wondered whether activation of ChrA is solely dependent on HrrS. Cross-talk between the kinases was previously described (Hentschel et al., 2014) and might explain the activation of the *hrtBA* operon in the evolved clone. To test this hypothesis, the *hrrS* gene was deleted in evolved strain 1.fs and compared to a Δ*hrrS* mutant in the parental background. Figure 4B shows that upon deletion of *hrrS*, 1.fs grew with an elongated lag phase of ∼50 h, which is even longer than for the WT. This result was in agreement with reporter assays showing that *hrrS* deletion also led to an abolishment of the *hrtBA* expression in 1.fs (Figure 4C). These results confirmed our hypothesis that HrrS is essential for activating *hrtBA* expression in the evolved clone encoding the catalytically-inactive pseudokinase of ChrS. Deletion of *hrrA* in the evolved variant did not influence its improved heme tolerance, therefore indicating that this is mainly an effect of HrrS activating ChrA (Supplementary Figure 5A,B). Overexpression of *chrA* also improved the heme tolerance to some extent (Supplementary Figure 5C). Interestingly, a plasmid-based overexpression of *hrtBA* in the WT background led to growth defects, probably caused by severe iron/heme starvation due to the excessive heme export (Supplementary Figure 6). This is further supported by comparative transcriptome analyses showing a higher upregulation in the strain overexpressing *hrtBA* compared to the evolved clone. This demonstrates the necessity of a tight balance between export and intracellular iron availability to maintain homeostasis while achieving optimal heme tolerance.

To investigate if the truncated version of ChrS still plays a role in the phosphotransfer to ChrA, we exchanged the conserved histidine residue at position 186 to an alanine (Figure 4D). This amino acid exchange did, however, not significantly influence the heme tolerance of strain 1.fs. Therefore, it can be assumed that the truncated version of ChrS does not participate in the phosphotransfer *via* autophosphorylation at the histidine 186.

Based on these results, we postulated that a lack of the catalytic activity of ChrS is beneficial for *C. glutamicum* heme tolerance. In line with this hypothesis, an in-frame deletion of the CA-domain of *chrS* led to improved growth on heme compared to the wild type. However, this strain did not reach comparably high heme tolerance like the 1.fs strain (Figure 4E). Upon additional deletion of the DHp-domain, this growth advantage was abolished suggesting the necessity of homo- or heterodimerization and/or interaction with ChrA.

### ChrS and HrrS form heterodimers *in vivo*

In general, HKs act as homodimers. However, based on the fact that the autophosphorylation histidine residue of ChrS in the evolved mutant 1.fs was not relevant for its growth benefit on heme (Figure 4D), we aimed to investigate the homo- and heterodimerization properties of the *C. glutamicum* HKs ChrS and HrrS and the pseudokinase ChrS (ChrS_CA-fs).

**FIGURE 5:**
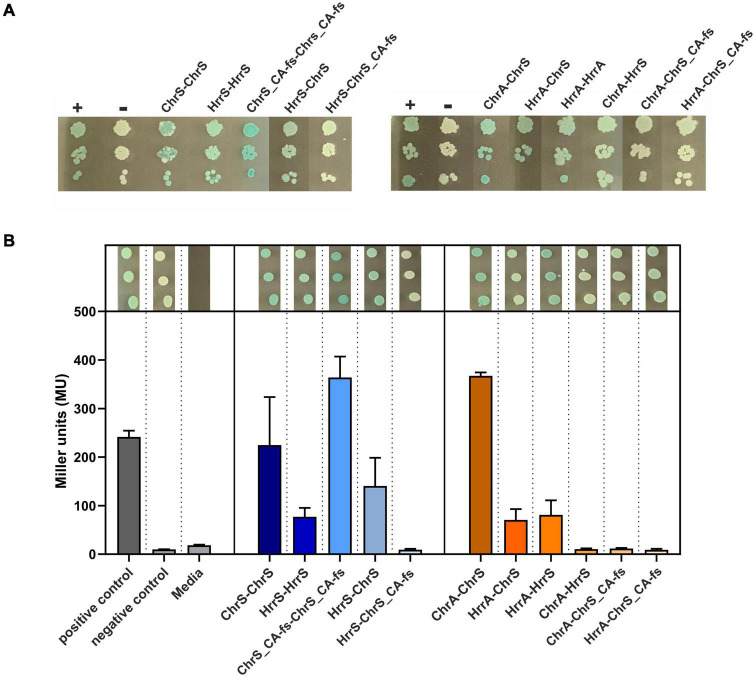
Bacterial two-hybrid assays of interactions between ChrSA and HrrSA. **(A)** BACTH interactions between the histidine kinases ChrS, HrrS and the evolved ChrS variant (here ChrS_CA-fs) were analyzed as C-terminal fusions; results for N-terminal variants are shown in Supplementary Figure 7. Blueish color of the colonies indicate interaction, while white colonies indicate no interaction (Euromedex, Souffelweyersheim, France). First histidine kinase represents the T25-fusion, the second the T18-fusion. + = pKTN25-zip with pUT18-zip (leucine zipper, positive control), – = pKTN25 with pUT18 (negative control). **(B)** Quantitative analysis using a β-galactosidase assay. Triplicates were cultivated and treated according to Griffith and Wolf (2002) to measure colorimetric β-galactosidase activity (given in Miller units). Gray bars represent the controls, blue bars show the interaction between the histidine kinases, and orange bars show the interaction of histidine kinase with the response regulators. Drop assays on top of the bar plots represent the triplicates picked after re-cultivation.

To assess these protein-protein interactions between the respective monomers ChrS, HrrS and the truncated HK variant ChrS_CA-fs, we performed bacterial two-hybrid (BACTH) assays (Euromedex, Souffelweyersheim, France). The plate assays in Figure 5A as well as the quantitative β-galactosidase assay in Figure 5B show that homodimerization for both the native and the evolved HKs was observed when these fusion proteins were produced as C-terminal fusions in *E. coli*. The assays also revealed heterodimerization of the native HKs, while there was no significant evidence for heterodimerization of the truncated ChrS_CA-fs version. Similar results were observed when the proteins were produced as N-terminal fusions (Supplementary Figure 7).

In a next set of experiments, we investigated the interaction between the sensor kinases and the RRs. Here, BACTH assays confirmed the interaction of ChrS with ChrA and HrrS with HrrA, as well as the cross-talk between ChrS and HrrA. Interaction was not observed for ChrS_CA-fs and neither ChrA nor HrrA within the β-galactosidase assay, although a slight signal appeared to be visible on plates (Figure 5). However, the assays did also not reveal the already reported cross-talk between HrrS and ChrA (Hentschel et al., 2014) demonstrating also the limitations of the *in vivo* approach based on the *E. coli* system. Therefore, a direct interaction between ChrA and the evolved ChrS_CA-fs in the *C. glutamicum in vivo* background should not be excluded.

### Heme-binding proteins contribute to heme tolerance

Within this study, we showed the crucial role of the HrtBA export system for *C. glutamicum* heme tolerance. To identify further potentially relevant factors, we performed a comparative transcriptome analysis of the ChrS-Ala245fs strain and *C. glutamicum* wild type (Table 2). As expected, the *hrtBA* operon showed significantly increased mRNA levels in the evolved clone (∼150-fold increase). Besides *hrtBA*, several other heme-related targets also showed increased expression levels, including the TCS *chrSA* itself, *hmuO* encoding heme oxygenase and the heme transport system *hmuU*. Quantitative PCR confirmed unaltered expression levels of *hrrS* (Supplementary Figure 4). Strikingly, all genes encoding known heme-binding proteins were significantly upregulated. Furthermore, many targets of the DtxR regulon were upregulated (Wennerhold and Bott, 2006), while targets of the RipA regulon were downregulated (Wennerhold et al., 2005). This indicates that the evolved clone encounters a strong iron depletion, most likely caused by the extreme heme export. This is in line with a growth defect of this strain under iron starvation conditions (Figure 3C).

**TABLE 2:** Comparative transcriptome analysis of *C. glutamicum* wild type and *C. glutamicum chrS*-Ala245fs growing on 4 μM heme.

Category and cg gene number	Gene designation and description of product	mRNA ratio^a^	*P-value*
**Heme-related genes**			
cg0468	*hmuU*, hemin transport system, permease protein	16.90	0.05
cg2200	*chrA*, two-component system, response regulator	8.85	0.01
cg2201	*chrS*, two-component system, signal transduction histidine kinase	43.60	0.04
cg2202	*hrtB*, ABC-type transport system, permease component	170.13	0.00
cg2204	*hrtA*, ABC-type transport system, ATPase component	151.54	0.00
cg2445	*hmuO*, heme oxygenase	8.30	0.01
**Heme-binding proteins**			
cg0466	*htaA*, secreted heme-transport associated protein	5.80	0.01
cg0467	*hmuT*, hemin-binding periplasmic protein precursor	21.23	0.06
cg0470	*htaB*, secreted heme transport-associated protein	66.64	0.02
cg0471	*htaC*, secreted heme transport-associated protein	16.87	0.04
cg3156	*htaD*, secreted heme transport-associated protein	18.64	0.05
**DtxR regulon**			
cg0160	Hypothetical protein cg0160	2.98	0.00
cg1120	*ripA*, transcriptional regulator of iron proteins, AraC family	5.76	0.10
cg1419	Putative Na^+^-dependent transporter	4.85	0.01
cg1476	*thiC*, thiamine biosynthesis protein ThiC	2.48	0.04
cg1695	Putative plasmid maintenance system antidote protein	0.34	0.05
cg1930	Putative secreted hydrolase	5.68	0.01
cg1930	Putative secreted hydrolase	5.68	0.01
cg1931	Putative secreted protein	9.98	0.05
cg1931	Putative secreted protein	9.98	0.05
cg2311	SAM-dependent methyltransferase	3.47	0.00
cg2444	Hypothetical protein cg2444	4.67	0.01
cg2782	*ftn*, ferritin-like protein	0.32	0.06
cg2796	MMGE/PRPD family protein	11.01	0.00
cg2962	Uncharacterized enzyme involved in biosynthesis of extracellular polysaccharides	6.69	0.02
**RipA regulon**			
cg0310	*katA*, catalase	0.14	0.00
cg0445	*sdhC*, succinate dehydrogenase	0.36	0.02
cg0446	*sdhA*, succinate dehydrogenase	0.40	0.00
cg0447	*sdhB*, succinate dehydrogenase	0.45	0.00
cg1343	*narH*, probable respiratory nitrate reductase oxidoreduct	0.49	0.03
cg1344	*narG*, nitrate reductase 2, alpha subunit	0.30	0.00
cg1487	*leuC*, isopropylmalate isomerase large subunit	0.29	0.01
cg1737	*acn*, aconitate hydratase	0.29	0.01
cg2636	*catA1*, catechol 1,2-dioxygenase	0.03	0.00
cg3048	*pta*, phosphate acetyltransferase	0.24	0.00

^a^Expression of selected genes given as the mRNA ratio of the evolved strain compared to the WT (>2-fold or < 0.5-fold, p-value < 0.05). Data represent the average of three biological replicates (for a complete list of up- and downregulated genes, see Supplementary Table 4).

**FIGURE 6:**
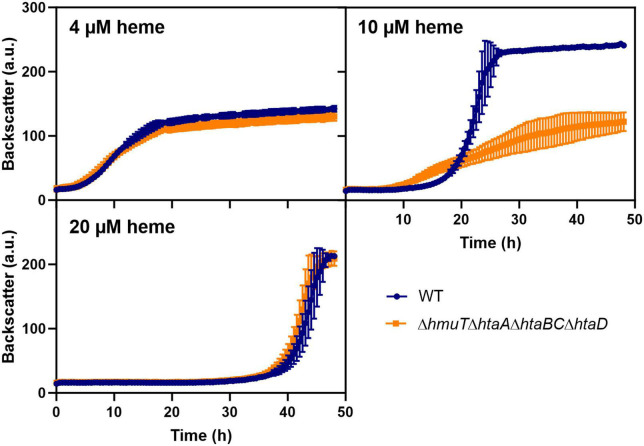
Heme binding proteins contribute to heme tolerance. The *C. glutamicum* wild type (WT) (blue) and the heme binding deficient mutant Δ*hmuT*Δ*htaA*Δ*htaBC*Δ*htaD* strain (orange) were inoculated at a starting-OD_600_ of 1 in CGXII medium containing 2% glucose and either 4, 10, or 20 μM heme. Data represent the average of three biological replicates including standard deviations depicted as error bars.

To test whether the upregulation of heme-binding proteins could also contribute to heme tolerance mediated by heme sequestration, we further analyzed the impact of heme binding proteins by the construction of serial deletions (Figure 6). A strain lacking the heme binding proteins *hmuT*, *htaA*, *htaB*, *htaC*, and *htaD* showed wild typic growth at low (4 μM) and high (20 μM) heme levels. However, at moderate (10 μM) heme concentrations, the mutant showed a significant growth defect. These results suggested that heme sequestration *via* heme-binding proteins could promote heme tolerance at moderate levels.

## Discussion

In this study, we pursued a laboratory evolution approach to adapt *C. glutamicum* ATCC 13032 to high heme levels. This ALE approach resulted in the isolation of strains harboring a frameshift mutation in the *chrS* HK gene yielding a catalytically inactive pseudokinase, which was shown to promote high heme tolerance of up to 100 μM. This effect could mainly be attributed to the strong upregulation of the heme exporter HrtBA and was strictly dependent on phosphotransfer *via* the non-cognate HK HrrS to the RR ChrA. Further mutational analysis confirmed that the conserved histidine residue of the ChrS pseudokinase (ChrS_CA-fs) was not involved in this phosphotransfer reaction.

Continuously high *hrtBA* expression levels observed in our evolved 1.fs strain are in agreement with a defect in ChrS phosphatase function of this strain. Remarkably, the evolved strains encoding the truncated pseudokinase variant ChrS showed significantly higher heme tolerance and higher *hrtBA* expression levels compared to a phosphatase deficient strain *chrS*-Q191A or a strain lacking *chrS* completely (Δ*chrS*) (Hentschel et al., 2014). Consequently, dephosphorylation of ChrA seems to be abolished in the mutant clones, but the presence of the ChrS pseudokinase is apparently further beneficial for enhancing heme tolerance.

Our results indicate that the catalytically inactive version of ChrS promotes—directly or indirectly—the efficient phosphotransfer reaction from the paralogous HrrS to ChrA leading to the constitutive activation of *hrtBA* (due to the absence of ChrS phosphatase activity). Gene duplication is a powerful evolutionary driving force and the presence of paralogs has previously been shown to be beneficial for adaptations to new environmental conditions (Gevers et al., 2004; Bratlie et al., 2010). In our study, the interaction between the two paralogous TCSs HrrSA and ChrSA enabled fast adaptation and the evolution of novel functionality of the signaling cascade based on the pseudokinase version of ChrS.

Pseudokinases are described as kinases lacking catalytic functions, but can contribute to signaling *via* functioning as allosteric modulators, dynamic scaffolds, or competitors of protein-protein interactions (Reiterer et al., 2014; Kwon et al., 2019; Tomoni et al., 2019). An example for a bacterial pseudokinase is DivL from *Caulobacter crescentus*. DivL controls the autophosphorylation of another HK CckA mediated by the phosphorylation status of the RR DivK. The direct interaction with DivL is required for maximal kinase activity of CckA, but this is achieved by a yet unknown mechanism (Iniesta et al., 2010; Tsokos et al., 2011; Francis and Porter, 2019). It was shown that neither the ATPase domain nor the autophosphorylation residue of DivL is necessary for its function (Reisinger et al., 2007)—which is similar to the scenario observed for ChrS in this study. It can therefore be hypothesized that the catalytically inactive ChrS pseudokinase has a stimulating effect on HrrS activity, e.g., by influencing it’s “on” or “off” states. An alternative or additional reason for enhanced phosphotransfer could also be the recruitment of ChrA *via* the ChrS pseudokinase fostering phosphotransfer from HrrS by the formation of heterodimers. In fact, heterodimerization between the native versions of ChrS and HrrS could be demonstrated using BACTH assays speaking for a direct signaling between ChrS and HrrS *in vivo*. However, interaction could not be observed with the truncated version of ChrS, but it has to be kept in mind that the *E. coli* based BACTH does not perfectly reflect the *C. glutamicum in vivo* situation. Here, we also observed high upregulation of the *chrSA* operon itself offering also enhanced levels of RR acceptor protein.

Although only a few reports on heterodimerization of HKs exist up to date (Capra and Laub, 2012; Willett and Crosson, 2017), studies in e.g., *Pseudomonas aeruginosa* showed that the HK RetS directly controls the HK GacS on three different levels by heterooligomerization (Goodman et al., 2009; Jing et al., 2010; Francis et al., 2018). Such pivotal (multiple) regulatory roles of heterodimers or –oligomers could also play a role in the native *C. glutamicum* system. In previous studies, HrrS was shown to act as “kickstarter” of the ChrSA-mediated response and the absence of HrrS led to a delayed promoter activation of *hrtBA* (Keppel et al., 2019). Consistently, heterooligomerization of HrrS with ChrS could support the fast activation of ChrA when heme becomes available, e.g., by cross-phosphorylation on the HK-level. An alternative option could be an indirect communication between ChrS and HrrS *via* another—yet unknown—component involved in cross-signaling (Buelow and Raivio, 2010; Salvado et al., 2012).

Apart from the conserved heme export system HrtBA, transcriptome analysis gave further hints for additional players contributing to heme tolerance in *C. glutamicum*. Here, we observed a high upregulation of all genes encoding heme-binding proteins and a deletion mutant showed reduced tolerance to intermediate heme levels. In fact, heme-binding proteins could serve a detoxifying role *via* heme sequestration, as described throughout the literature for other organisms, including HbpC of *Bartonella henselae* (Roden et al., 2012), HemS of *Yersinia enterocolitica* (Stojiljkovic and Hantke, 1994) or HupZ of Group A *Streptococcus*, which has been recently hypothesized to function as heme chaperone (Lyles et al., 2022).

Moreover, several transport systems are differently expressed in the evolved strains. Export of further toxic heme-related products, or even import of neutralizing compounds coping with H_2_O_2_, like e.g., described for the ribulose-5-phosphate 3-epimerase in *Escherichia coli* or the Mn(II) uptake system of *Neisseria gonorrhoeae* importing manganese (Horsburgh et al., 2002; Seib et al., 2004; Sobota and Imlay, 2011), could also aid at tolerating heme. Of special interest is e.g., the operon cg2675-cg2678, which encodes an ABC-type transport system and was found also to be regulated by the heme-responsive RR HrrA (Keppel et al., 2020). Future studies on these systems, are however, required to elucidate their role in heme tolerance or homeostatic processes.

The appearance of a ChrS pseudokinase was not yet described to occur naturally in corynebacterial strains (Bott and Brocker, 2012). However, our experimental approach demonstrated their ability to quickly adapt to high heme levels, which is for example relevant for virulence conditions in the mammalian host (Stauff and Skaar, 2009a). In the soil environment, a constant expression of the *hrtBA* operon is likely too costly as reflected by the strong upregulation of the iron starvation response in the evolved clones, including the regulon of DtxR (Wennerhold and Bott, 2006) and the downregulation of RipA targets (Wennerhold et al., 2005). The native cascade consequently rather facilitates a fast but transient activation of *hrtBA* in response to heme levels (Keppel et al., 2019).

Strains featuring an elevated heme tolerance are also highly interesting for the biotechnological production of heme, which is commercially produced for medical uses or the food sector for artificial meat products. Recent metabolic engineering efforts resulted in *E. coli* (Kwon et al., 2003; Zhao et al., 2018) and *C. glutamicum* heme-producing strains (Ko et al., 2021) achieving heme yields of about 0.14–0.61 mmol mol^–1^. In this context, the pseudokinase variant of ChrS described in this study might aid future metabolic engineering approaches to promote efficient heme export and high product tolerance.

## Data availability statement

The datasets presented in this study can be found in online repositories. The names of the repository/repositories and accession number(s) can be found in the article/Supplementary material.

## Author contributions

JF and AK conceived and designed the analysis and wrote the manuscript. AK performed the experiments and collected the data, contributed to data and analysis tools, and performed the analysis. Both authors contributed to the article and approved the submitted version.
