# Supplementary material for: A pseudokinase version of the histidine kinase ChrS promotes high heme tolerance of Corynebacterium glutamicum
Source: Front Microbiol. 2022 Sep 7;13:997448. doi: 10.3389/fmicb.2022.997448 (PMC9491836; doi:10.3389/fmicb.2022.997448)
Supplement: Supplementary file 1 [file Data_Sheet_1.pdf]

Supplemental material to: A pseudokinase version of the histidine kinase ChrS promotes high heme tolerance of *Corynebacterium glutamicum*

This file includes:

Figure S1: Adaptive laboratory evolution of *C. glutamicum* to high heme levels.

Figure S2: The ChrS-Ala245fs pseudokinase promotes heme tolerance.

Figure S3: HrtB-reporter assay.

Figure S4: qPCR analysis comparing expression levels of *hrtB* and *hrrS* in the *C. glutamicum* WT in comparison to the evolved strain.

Figure S5: ChrA is crucial for the activation of *hrtBA*, while *hrrA* does not affect the growth advantage of the evolved clone.

Figure S6: Plasmid-based overexpression of *hrtBA* does not lead to the same heme tolerance as in the evolved clone 1.fs due to too excessive heme export

Figure S7: BACTH with N-terminal tagged variants of the HKs ChrS and HrrS.

Table S1: Bacterial strains used within this study.

Table S2: Oligonucleotides used in this study.

Table S3: Plasmids used within this study

Table S4: Comparative transcriptome analysis of *C. glutamicum* WT and *C. glutamicum* ChrS-Ala245fs during growth on 4 µM heme.

Figures

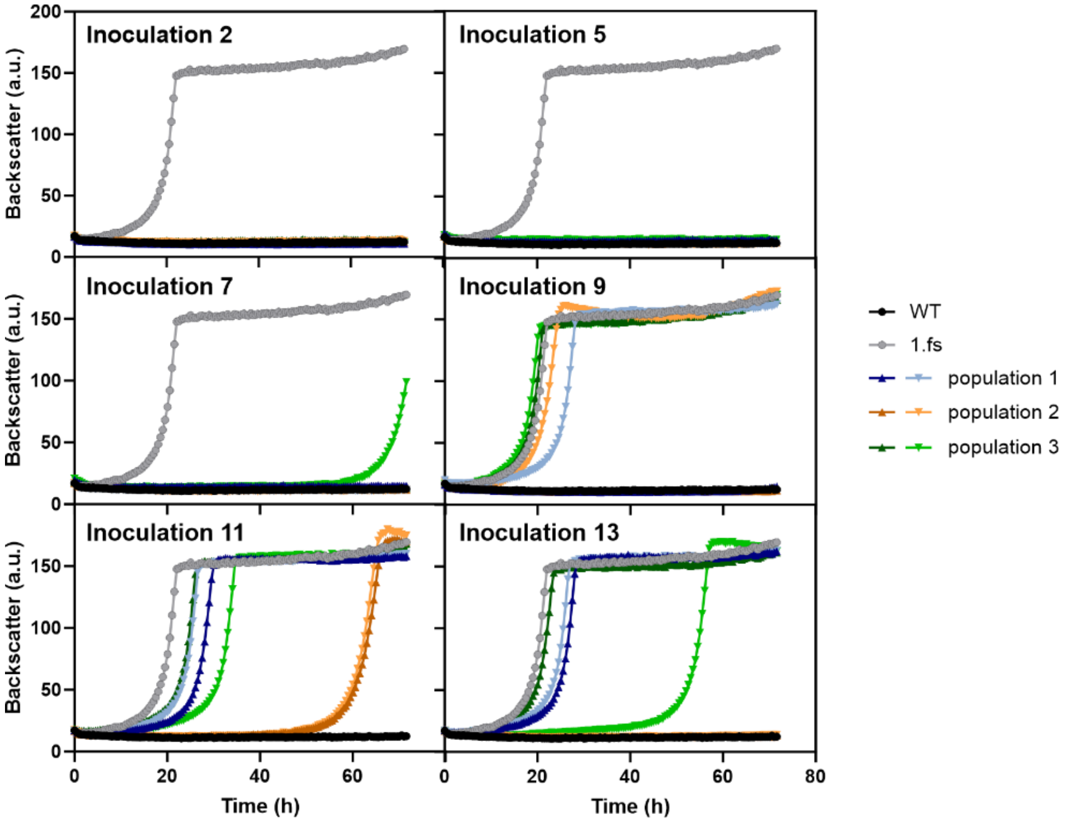

Figure S1: Adaptive laboratory evolution of *C. glutamicum* to high heme levels. *C. glutamicum* ATCC 13032 (WT, black) as well as the evolved strain (1.fs, grey) and each two single clones of the different populations 1 to 3 (blue, orange, green) from the respective inoculation steps in the ALE experiment were inoculated at a starting-OD_{600} of 1 in CGXII medium containing 2% glucose and 100 µM heme.

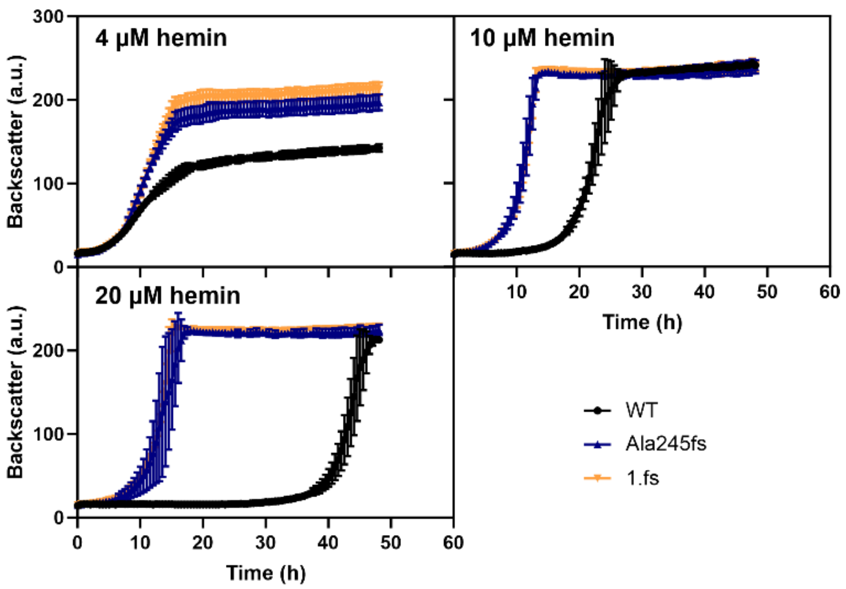

Figure S2: The ChrS-Ala245fs pseudokinase promotes heme tolerance. Data represent the averages of three biological replicates including standard deviations. Reintegration of the point mutation Ala245fs in in the parental *chrS* (blue) gene shows that this single mutation leads to enhanced heme tolerance of the evolved clone 1.fs (orange) compared to the WT (black) under all tested conditions.

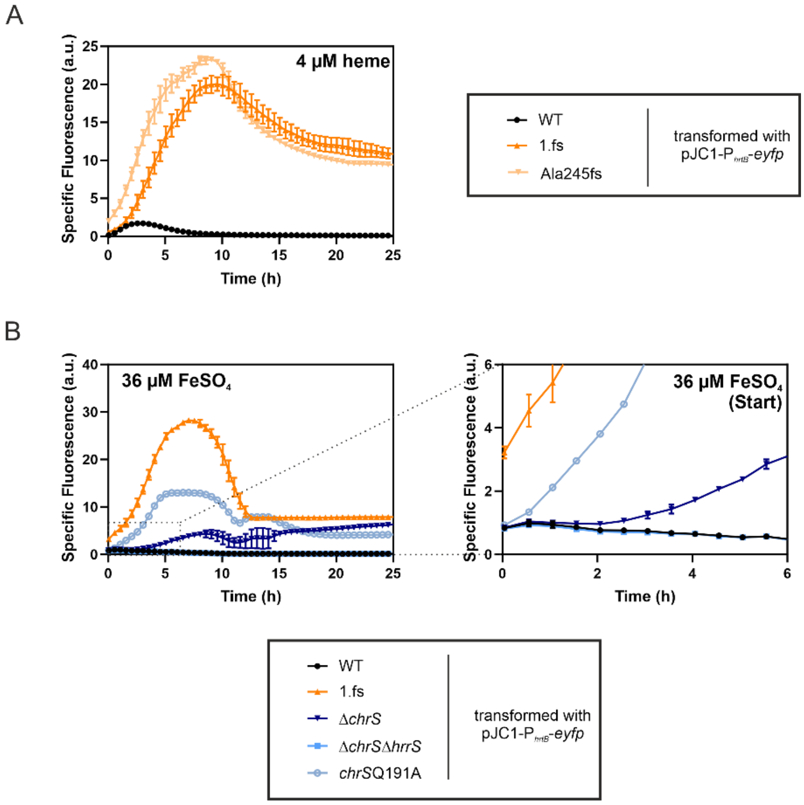

Figure S3: HrtB-reporter assay. Data represent the averages for three biological replicates including standard deviations as error bars. Cells were inoculated at a starting-OD_{600} of 1 in CGXII medium containing 2% glucose and the indicated amount of heme or iron. (A) Reporter assays for *hrtB*-expression using the plasmid pJC1-P$_{hrtB}$-*eyfp* for transformation of the WT (black), evolved clone 1.fs (orange) and the reintegration strain with Ala245fs in ChrS (light orange). The *hrtB* expression for the evolved strain 1.fs and the reintegration strain ChrS-Ala245fs is comparable in the presence of 4 µM heme. (B) Reporter assays for *hrtB*-expression using the plasmid pJC1-P$_{hrtB}$-*eyfp* for transformation of the WT (black), evolved clone 1.fs (orange), Δ*chrS* (dark blue), Δ*chrS*Δ*hrrS* (mid-blue) and *chrS*-Q191A (light blue). 1.fs shows an extreme high upregulation of the heme exporter even at standard conditions (36 µM $FeSO_4$, no presence of added heme).

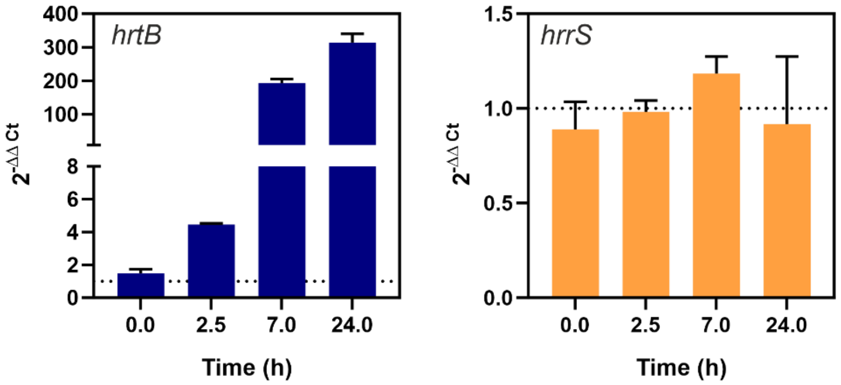

Figure S4: qPCR analysis comparing expression levels of *hrtB* and *hrrS* in the *C. glutamicum* WT in comparison to the evolved strain. The *C. glutamicum* WT and the imitated strain ChrS-Ala245fs were cultivated in 50 ml CGXII medium with 2% glucose and 4 µM heme in 500 ml shaking flasks. After 0, 2.5, 7 and 24 h, a sample was taken in ice-falcons for RNA isolation via the Monarch Total RNA Miniprep Kit (New England BioLabs, Frankfurt am Main). Using the Luna One-Step RT-qPCR Kit (New England BioLabs, Frankfurt am Main) according to manufacturer's instructions, qPCR was performed in the qTower (Analytik Jena, Jena) and analyzed using qPCRsoft 3.1 (Analytik Jena, Jena). As a reference gene for normalization, the housekeeping gene *ddh* (A156-*ddh*-qPCR-fw CCGGAAAGCAAACCCACAAG; A157-*ddh*-qPCR-rv CTCGGAGTCGAAGGTTGCTT) was used (Frunzke et al., 2008), besides the target genes *hrtB* (A269-*hrtB*-qPCR-fw TCCGATTTAGCCTCACTCGC; A270-*hrtB*-qPCR-rv AGTGACATCTGTTCGCCCTG) or *hrrS* (A463_*hrrS*_qPCR_fw AGTTCAACCTCGCCTGTAGC; A464_*hrrS*_qPCR_rv GATGGAATTGCACGGCGAAG). Data represent two biological and three technical replicates. Fold-change was calculated according to the $2^{-\Delta\Delta Ct}$ (Livak and Schmittgen, 2001).

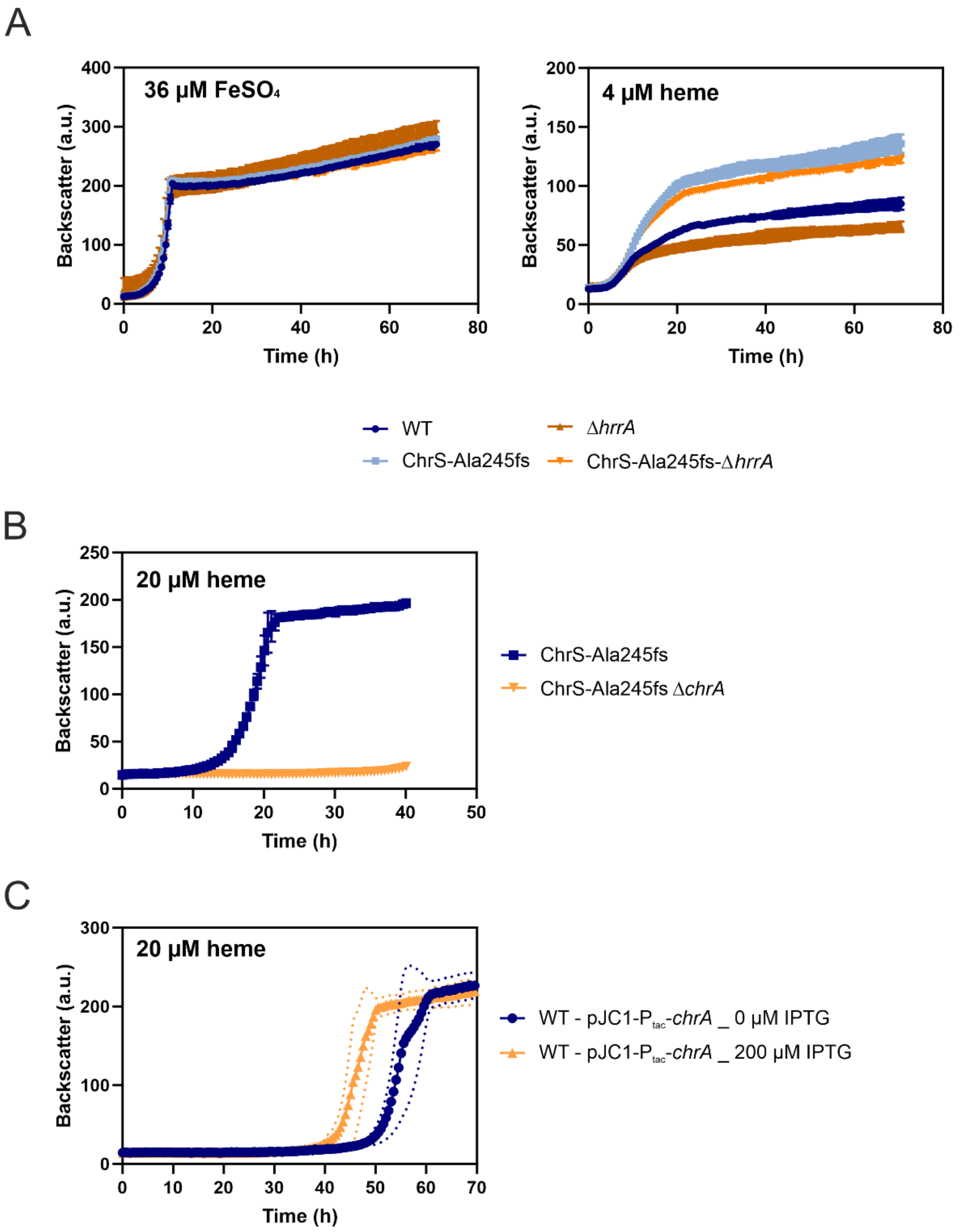

Figure S5: ChrA is crucial for the activation of *hrtBA*, while HrrA does not affect the growth advantage of the evolved clone. Cells were inoculated at a starting-OD_{600} of 1 in CGXII medium containing 2% glucose and the indicated amount of heme or iron. (A) *C. glutamicum* WT (dark blue), the strain encoding the pseudokinase ChrS-Ala245fs (light blue) and strains carrying an additional deletion of *hrrA* (shades of orange) were cultivated under standard conditions (36 µM $FeSO_4$) and with 4 µM heme. The deletion of *hrrA* does not significantly affect the growth advantage of the strain encoding the ChrS pseudokinase. (B) Deletion of *chrA* abolished the growth of the evolved clone on heme. (C) The WT strain was transformed with the overexpression plasmid pJC1-P_{tac}-*chrA* and cultivated with 20 µM heme and either without (blue) or with 200 µM IPTG (orange) for induction. Upon *chrA* overexpression, the strain shows slightly increased heme tolerance. Data represent the averages of three biological replicates including standard deviations.

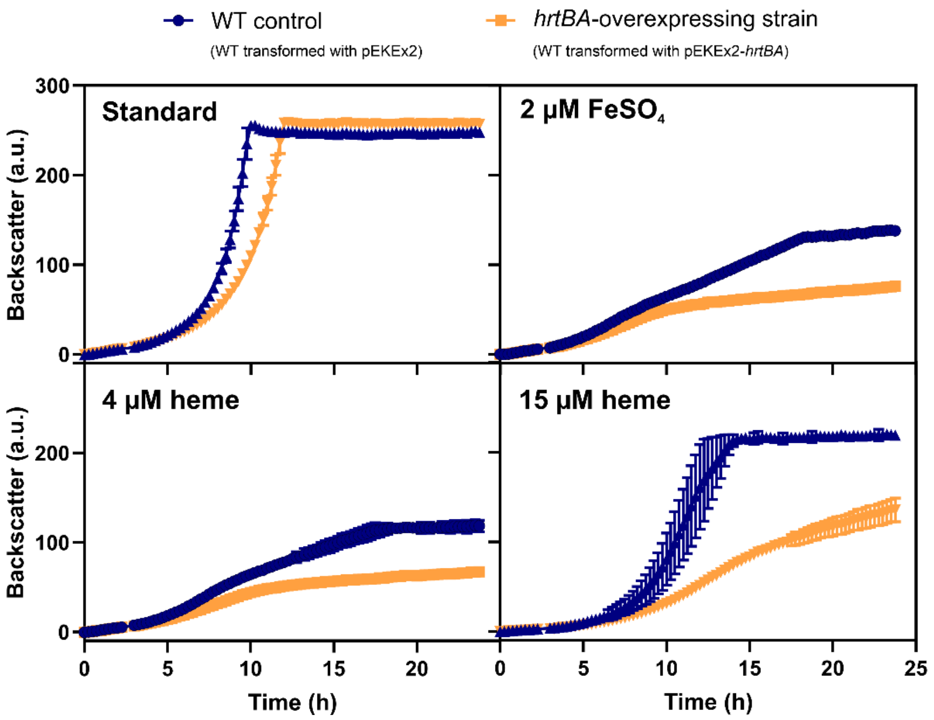

Figure S6: Plasmid-based overexpression of *hrtBA* does not lead to the same heme tolerance as in the evolved clone 1.fs due to too excessive heme export. *C. glutamicum* WT was transformed with an empty plasmid pEKEx2 (Eikmanns et al., 1994) (blue) and a *hrtBA* overexpressing plasmid pEKEx2-*lacI*-P_{tac}-*hrtBA* (Heyer et al., 2012) (orange). Cells were inoculated at a starting-OD_{600} of 1 in CGXII medium containing 2% glucose, 25 µg/ml kanamycin, 100 µM IPTG for induction and the indicated amount of heme or iron. Standard refers to 36 µM $FeSO_4$. The overexpression of *hrtBA* did lead to a growth defect in every condition tested, probably caused by too excessive heme export leading to a severe iron starvation phenotype. As comparison, microarrays of WT compared to the evolved clone presented in this study showed a ~150-fold upregulation of *hrtBA* (p-value: 0.002), while this overexpressing strain shows a ~400-fold upregulation (p-value: 0.002) (data not shown).

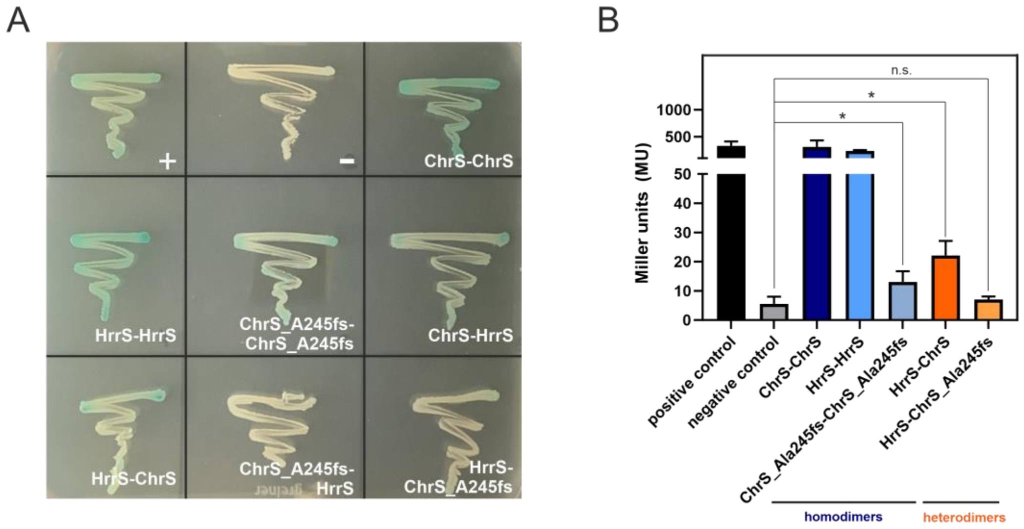

Figure S6: BACTH with N-terminal tagged variants of the HKs ChrS and HrrS. (A) Interactions between the histidine kinases ChrS, HrrS and the ChrS pseudokinase variant (here ChrS_Ala245fs). Blueish color of the colonies indicates a direct interaction, while white represents no interaction. The first HK represents a T25-fusion, the second a T18-fusion. + = pKTN25-zip with pUT18-zip (leucine zipper, positive control), - = pKTN25 with pUT18 (negative control). (B) After transformation, cells were plated, and for each combination, three single colonies were picked for further quantitative analysis using a β-galactosidase assay. Triplicates were re-cultivated overnight and treated according to Griffith and Wolf (2002) to measure colorimetric β-galactosidase activity. Miller units were calculated and are represented in this bar graph. Black and grey bars represent the controls, blue bars show homodimer interactions, and orange bars show heterodimer interactions. Significance was evaluated by an unpaired t-test with a 95% confidence interval.

Tables

Table S1: Bacterial strains used in this study.

Strain	Characteristics	Reference
C. glutamicum ATCC 13032	Wild type (WT), biotin auxotroph	Kinoshita et al. (2004)
Δ*hrtBA*	ATCC 13032 derivative with deletion of *hrtBA*	Heyer et al. (2012)
1.fs	ATCC 13032 evolved on heme, frameshift mutant, chrS-730delG	This work
chrS-Ala245fs	ATCC 13032 derivative with 730delG leading to frameshift at Ala245, Imitation of 1.fs	This work
Δ*chrS*	ATCC 13032 derivative with deletion of *chrS*	Hentschel et al. (2014)
Δ*chrS*Δ*hrrS*	ATCC 13032 derivative with deletion of *chrS* and *hrrS*	Hentschel et al. (2014)
chrS-Q191A	ATCC 13032 derivative with amino acid exchange Q191A in *chrS*, phosphatase mutant	Hentschel et al. (2014)
Δ*hrrS*	ATCC 13032 derivative with deletion of *hrrS*	Hentschel et al. (2014)
1.fs Δ*hrrS*	Evolution clone 1.fs derivative with deletion of *hrrS*	This work
chrS-H186A	ATCC 13032 derivative with amino acid exchange H186A in *chrS*, affects autophosphorylation histidine	This work
1.fs *chrS*-H186A	Evolution clone 1.fs derivative with amino acid exchange H186A in *chrS*, affects autophosphorylation histidine	This work
Δ*chrS*_DHp-CA	ATCC 13032 derivative with deletion of the whole DHp and CA domain of *chrS*	This work
Δ*chrS*_CA	ATCC 13032 derivative with deletion of the whole DHp and CA domain of *chrS*	This work
Δ*hmuT*Δ*htaA*Δ*htaBC*Δ*htaD*	ATCC 13032 derivative with deletion of *hmuT*, *htaA*, *htaBC* and *htaD*	This work
E. coli DH5α	F^- φ80lacZΔM15 Δ(lacZYA-argF)U169 recA1 endA1 hsdR17(rK^- mK^+) phoA supE44 thi-1 gyrA96 relA1 $λ^-$; for general cloning purposes	Invitrogen
E. coli BTH101	F^-, cya-99, araD139, galE15, galK16, rpsL1 (Str^r), hsdR2, mcrA1, mcrB1; reporter strain for BACTH assay	Euromedex

Table S2: Oligonucleotides used in this study.

Oligonucleotide	Sequence 5' → 3'	Use
**Construction of deletionplasmid pK19-mobsacB-*chrS*-Ala245fs**		
A250-chrS-730delG-lf_fw	**CAGGTCGACTCTAGAGGATC**GACCGGAGCCTGCGTATTTGG	Left flank
A251-chrS-730delG-lf-rv	CGGCAGATCCGCCCAATGAG	
A252-chrS-730delG-rf-fw	CTCATTGGGCGGATCTGCCG	Right flank
A253-chrS-730delG-rf-rv	**GTAAAACGACGGCCAGTGAATTG**CATCATCTGGGGTGCCCACC	
A260-chrS-seq	GGATGATCCGCAGGCGTTAAGC	Sequencing *chrS* region
**Construction of deletionplasmid pK19-mobsacB-Δ*hrrS***		
A303-DhrrS-leftflank_fw	**CAGGTCGACTCTAGAGGATC**GATTGGTGAACACGATGACCGCG	Left flank
A304-DhrrS-leftflank-rv	GTCTGTAACCGAGCATCTCTC**CTA**CTAGGCTTGACTGCATGCGTCG	
A305-DhrrS-rightflank-fw	GAGAGATGCTCGGTTACAGACGGCGAGGTTGAACTAAGTTCTCC	Right flank
A306-DhrrS-rightflank-rv	**GTAAAACGACGGCCAGTGAATTG**GGGTGACAGTGTGGAGTCACC	
A307-DhrrS-seq3-fw	CCATGGGTGTCCACACTGATG	Sequencing of *hrrS* region
A308-DhrrS-seq4-rv	GTTCACGTGGGGTGAGTGAGG	
**Construction of plasmid pK19-mobsacB-*chrS*-H186A for amino acid exchange**		
A322-chrS-H186A-lf_fw	**CAGGTCGACTCTAGAGGATC**GACCATCGCCCGAATTGAGAGAG	Left flank
A323-chrS-H186A-lf-rv	CTGCGCCACAGTGTCGGCTATTTCGCCCGCTATGCG	
A324-chrS-H186A-rf-fw	CGCATAGCGGGCGAAATAGCCGACACTGTGGCGCAG	Right flank
A325-chrS-H186A-rf-rv	**GTAAAACGACGGCCAGTGAATTG**CGGAAATTCCAGTTCCCGATCCG	
A326-chrS-HIS-seq_fw	GTACGACCAAAGTCGGATTCGCG	Sequencing of *chrS* region
A327-chrS-HIS-seq_rv	GCGCGAACAACGGGATGATCATC	
A328-chrS-HISclose-seq_rv	CTCCGCCAAATTATCAGCTGTCG	
**Construction of deletionplasmids for the deletion of ChrS regions**		
A298-DchrS-CAdomain-leftflank_fw	**CAGGTCGACTCTAGAGGATC**CACCGCTGAAATCCGCGATCATC	Left flank for deletion of CA-domain
A299-DchrS-CAdomain-leftflank-rv	GTCTGTAACCGAGCATCTCTC**CTA**TCTGCCTCCATCGCATCAGGTAA	
A300-DchrS-CAdomain-rightflank-fw	GAGAGATGCTCGGTTACAGACCCGCCCGTTTTCCCTATCCAC	Right flank for deletion of CA-domain
A301-DchrS-CAdomain-rightflank-rv	**GTAAAACGACGGCCAGTGAATTG**CGCCACCAAGGACAGCACTTC	
A314-DchrS-DHp-leftflank_fw	**CAGGTCGACTCTAGAGGATC**CTAGCCAAGCGACCATCGCC	Left flank for deletion of DHp- and CA-domain
A315-DchrS-DHp-leftflank-rv	GTCTGTAACCGAGCATCTCTC**CTA**TGCCTGCTCGCCAGCGCTTT	
A300-DchrS-CAdomain-rightflank-fw	GAGAGATGCTCGGTTACAGACCCGCCCGTTTTCCCTATCCAC	Right flank for deletion of DHp- and CA-domain
A301-DchrS-CAdomain-rightflank-rv	**GTAAAACGACGGCCAGTGAATTG**CGCCACCAAGGACAGCACTTC	
A265-ChrS-CA-fw	CAGTGTTGATCACGACACCGC	Sequencing of *chrS*
A266-ChrS-CA-rv	GACTTTTCACCGTGGCCTCAG	
**Construction of plasmids for deletion of heme binding proteins**		
A160-htaA-leftflank_fw	**CAGGTCGACTCTAGAGGATC**CCATGTGGCGTCGACGAACC	Left flank for deletion of
A161-htaA-leftflank-rv	GTCTGTAACCGAGCATCTCTC**CTA**GCTCCTCATCCGCATACGAGG	

		htaA and *hmuT*
A185-hmuT-rightflank-fw	GAGAGATGCTCGGTTACAGACGTCGCCGCAGCGTTCCATTGC	Right flank for deletion of *htaA* and *hmuT*
A186-hmuT-rightflank-rv	**GTAAAACGACGGCCAGTGAATTG**CACAACCATCTACTCGCCGCC	
A051-seq-hmuhtaA-2-fw	GTGTCTGAGCCATGGGATCGC	Sequencing of *htaA* and *hmuT*
A188-hmuT-seq-rv	CGGTCCGACGCTTAGCTATAGC	
A165-htaB-leftflank_fw	**CAGGTCGACTCTAGAGGATC**GTCCCGTGTCCTTGCTCAGC	Left flank for deletion of *htaB* and *htaC*
A166-htaB-leftflank-rv	GTCTGTAACCGAGCATCTCTC**CTA**GTGAGAGCAACGAGCGCACG	
A173-htaC-rightflank-fw	GAGAGATGCTCGGTTACAGACGCTGAGTGGTTCTACAGGTCGG	Right flank for deletion of *htaB* and *htaC*
A174-htaC-rightflank-rv	**GTAAAACGACGGCCAGTGAATTG**GAAGCCGCACAGTAAAGAGCGC	
A169-htaB-seq-fw	CCGCCACCTCGGTATCGCAC	Sequencing of *htaB* and *htaC*
A176-htaC-seq-rv	GCATCAACGTCACCCTGACCG	
A177-htaD-leftflank_fw	**CAGGTCGACTCTAGAGGATC**GTTCGATTCCCGTCACCGGC	Left flank for deletion of *htaD*
A178-htaD-leftflank-rv	GTCTGTAACCGAGCATCTCTC**CTA**CAGCGGTCAGTACTGCGAAAGC	
A179-htaD-rightflank-fw	GAGAGATGCTCGGTTACAGACGGGCCAGCTGAACTACGAAGG	Right flank for deletion of *htaD*
A180-htaD-rightflank-rv	**GTAAAACGACGGCCAGTGAATTG**CCTTCACCAAACGTGGTCTGC	
A181-htaD-seq-fw	GCGCCACCTTTTACACTGCCG	Sequencing of *htaD*
A182-htaD-seq-rv	CCAGAGAAACCGTCACCACGG	
**Sequencing primers for pK19-constructs**		
M13_fw	CGCCAGGGTTTTCCCAGTCAC	Sequencing of plasmid
M13_rv	AGCGGATAACAATTTCACACAGGA	
**Construction of plasmid for overexpression of chrA**		
J545-lacI-Ptac_fw	**AGCGACGCCGCAGGGGGATCC**TCAAGCCTTCGTCACTGGTCCC	P_{tac} and *lacI* as inducible promoter
J546-lacI-Ptac_rv	ATGATATCTCCTTCTTAAAGTTCAGGATCCTCTAGAGTCGACCTGC	
A523-ChrA_oex_fw	TGAACTTTAAGAAGGAGATATCATGTGATCCGTATTCTGTTGGCTG	*chrA* insert
A524-ChrA-oex_rv	**AAAACGACGGCCAGTACTAG**CTAGATAATTCCGCGCTGTCTG	
**Sequencing primers for pJC1-constructs**		
pJC1-MCS-fw	CAGGGACAAGCCACCCGCACA	Sequencing of plasmid
pJC1-MCS-rv	GGAAGCTAGAGTAAGTAGTTCGC	
**Bacterial two-hybrid assays**<br>Note: for pKTN25 and pUT18 constructs, the same primers were used only backbone varied		
A380-BACTH-NT25-ChrS-lf_fw	**GCATGCCTGCAGGTCGACT**GTGAAAACTAGCCAAGCGACCA	Insert ChrS and ChrS_CA-fs -> Differing Template
A381-BACTH-NT25-ChrS-rf_rv	**GCTCGGTACCCGGGGATC**CTTATCTTGGTCCTTTTGTGGATAG	
A382-BACTH-NT25-HrrS-lf_fw	**GCATGCCTGCAGGTCGACT**ATGCAGTCAAGCCTAGATCG	Insert HrrS
A383-BACTH-NT25-HrrS-rf_rv	**GCTCGGTACCCGGGGATC**CTCATCGTCAGTTGGAGAACTTAG	
A455-BACTH-NT25-ChrA-lf_fw	**GCATGCCTGCAGGTCGACT**GTGATCCGTATTCTGTTGGCTG	Insert ChrA
A456-BACTH-NT25-ChrA-rf_rv	**GCTCGGTACCCGGGGATC**GATAATTCCGCGCTGTCTGGCT	
A457-BACTH-NT25-HrrA-lf_fw	**GCATGCCTGCAGGTCGACT**ATGATTCGCGTGCTGCTTGC	Insert HrrA
A458-BACTH-NT25-HrrA-rf_rv	**GCTCGGTACCCGGGGATC**CAGCAGCCCCTGCTCACGTG	

A357-pUT18seq_fw	GGTGTCGGGGCTGGCTTAAC	Sequencing pKTN25 constructs
M13_rv	AGCGGATAACAATTTCACACAGGA	
M13_fw	CGCCAGGGTTTTCCCAGTCAC	Sequencing pUT18 constructs
M13_rv	AGCGGATAACAATTTCACACAGGA	

Bold = overlap to backbone; orange = overlap of inserts

Table S3: Plasmids used in this study.

Plasmid	Characteristics	Reference
pK19-*mobsacB*	Contains negative (*sacB*) and positive (Kan^r) selection markers for genomic integration and deletion, MCS cut with EcoRI/BamHI	Schäfer et al. (1994)
pK19-*mobsacB-chrS*-Ala245fs	Derivative of pK19*mobsacB* deletion of a single guanine at position 730 in chrS, leading to a frameshift after Ala245; Kan^r	This work
pK19-*mobsacB*-Δ*hrrS*	Derivative of pK19*mobsacB* for partial *hrrS* gene deletion; Kan^r	Hentschel et al. (2014)
pK19-*mobsacB-chrS*-H186A	Derivative of pK19*mobsacB* for amino acid exchange at the autophosphorylation histidine (H186A) in *chrS*; Kan^r	This work
pK19-*mobsacB*-Δ*chrS*_DHp-CA	Derivative of pK19*mobsacB* for partial deletion of *chrS*, deletion of DHp and CA-domain; Kan^r	This work
pK19-*mobsacB*-Δ*chrS*_CA	Derivative of pK19*mobsacB* for partial deletion of *chrS*, deletion of CA-domain Kan^r	This work
pK19-*mobsacB*-Δ*chrA*	Derivative of pK19*mobsacB* for partial *chrA* gene deletion; Stop Codon included; Kan^r	This work
pK19-mobsacB- Δ*hrrA*	Derivative of pK19*mobsacB* for partial *hrrA* gene deletion; Stop Codon included; Kan^r	(Frunzke et al., 2011)
pJC1-*venus*-term-BS	*E. coli-C. glutamicum* shuttle vector; derivative of pJC1 (Cremer et al., 1990), containing the terminator sequence of *Bacillus subtilis* behind venus; $oriV_{Ec}$ $oriV_{Cg}$; Kan^r	Baumgart et al. (2013)
pJC1-P_{hrtB}-*eyfp*	Derivative of pJC1-venus-term-BS, containing *eyfp* under the control of the promoter P_{hrtB}	Heyer et al. (2012)
pJC1-P_{tac}-*chrA*	Derivative of pJC1-venus-term-BS, containing *chrA* under the control of the promoter P_{tac} and lacI	This work
pKTN25	C-terminal T25 protein fusion plasmid, pSU40 derivative, MCS cut with XbaI/BamHI; Kan^r	Euromedex
pUT18	C-terminal T18 protein fusion plasmid, pUC19 derivative, MCS cut with XbaI/BamHI; Amp^r	Euromedex
pKT25-zip	Derivative of pKT25, leucine zipper of GCN4 fused to T25, serves as positive control; Kan^r	Euromedex
pUT18C-zip	Derivative of pUT18C, leucine zipper of GCN4 fused to T18, serves as positive control; Amp^r	Euromedex
pKTN25-*chrS*	Derivative of pKT25 for ChrS with T25 N-terminally fused; Kan^r	This work
pKTN25-*chrS*_CA-fs	Derivative of pKT25 for ChrS-Ala245fs (evolution variant) with T25 N-terminally fused; Kan^r	This work
pKTN25-*hrrS*	Derivative of pKT25 for HrrS with T25 N-terminally fused; Kan^r	This work
pUT18-*chrS*	Derivative of pUT18C for ChrS with T18 N-terminally fused; Amp^r	This work
pUT18-*chrS*_CA-fs	Derivative of pUT18C for ChrS-Ala245fs (evolution variant) with T18 N-terminally fused; Amp^r	This work
pUT18-*hrrS*	Derivative of pUT18C for HrrS with T18 N-terminally fused; Amp^r	This work
pKTN25-*chrA*	Derivative of pKT25 for ChrA with T25 N-terminally fused; Kan^r	This work

pUT18-*chrA*	Derivative of pUT18 for ChrA with T18 N-terminally fused; Amp^r	This work

Table S4: Comparative transcriptome analysis of *C. glutamicum* WT and *C. glutamicum chrS*-Ala245fs during growth on 4 µM heme.

Gene	Average	*p*-value	Annotation
cg2202	170.13	0.00	*hrtB*, ABC-type transport system, permease component
cg2204	151.54	0.00	*hrtA*, ABC-type transport system, ATPase component
cg0470	66.64	0.02	*htaB*, secreted heme transport-associated protein
cg2201	43.60	0.04	*cgtS8*, two-component system, signal transduction histidine kinase
cg0467	21.23	0.06	*hmuT*, hemin-binding periplasmic protein precursor
cg3369	21.13	0.00	Rieske-type iron-sulfur protein
cg3156	18.64	0.05	*htaD*, secreted heme transport-associated protein
cg0468	16.90	0.05	*hmuU*, hemin transport system, permease protein
cg0471	16.87	0.04	*htaC*, secreted heme transport-associated protein
cg0771	15.90	0.05	secreted siderophore-binding lipoprotein
cg1418	12.64	0.06	secreted siderophore-binding lipoprotein
cg2796	11.01	0.00	MMGE/PRPD family protein
cg1931	9.98	0.05	putative secreted protein
cg2200	8.85	0.01	*cgtR8*, two-component system, response regulator
cg2445	8.30	0.01	*hmuO*, heme oxygenase | *hmuO*, heme oxygenase
cg2962	6.69	0.02	uncharacterized enzyme involved in biosynthesis of extracellular polysaccharides
cg0466	5.80	0.01	*htaA*, secreted heme-transport associated protein
cg1120	5.76	0.10	*ripA*, transcriptional regulator of iron proteins, AraC family
cg0927	5.76	0.02	siderophore ABC transporter, permease protein
cg0924	5.75	0.01	secreted siderophore-binding lipoprotein
cg1930	5.68	0.01	putative secreted hydrolase
cg3404	5.24	0.00	secreted siderophore-binding lipoprotein
cg0926	5.05	0.02	siderophore ABC transporter, permease protein
cg1419	4.85	0.01	putative Na^+-dependent transporter | putative Na^+-dependent transporter
cg2444	4.67	0.01	hypothetical protein cg2444 | hypothetical protein cg2444
cg0360	4.43	0.02	putative phosphatase
cg0590	4.24	0.00	siderophore ABC transporter, permease protein
cg0922	4.19	0.12	secreted siderophore-binding lipoprotein | secreted siderophore-binding lipoprotein
cg0768	4.08	0.00	siderophore ABC transporter, ATP-binding protein
cg2311	3.47	0.00	SAM-dependent methyltransferase
cg0589	3.42	0.03	siderophore ABC transporter, ATP-binding protein
cg2283	3.40	0.02	hypothetical protein cg2283
cg2678	3.33	0.13	ABC-type dipeptide/oligopeptide/nickel transport systems, secreted component
cg2061	3.16	0.00	*psp3*, putative secreted protein
cg0769	3.07	0.00	siderophore ABC transporter, permease protein
cg1327	3.06	0.03	bacterial regulatory proteins, Crp family
cg2199	3.05	0.08	pbp, penicillin-binding protein, putative D-alanyl-D-alanine carboxypeptidase
cg3219	3.03	0.01	*ldhA*, NAD-dependent L-lactate dehydrogenase
cgr05	3.02	0.05	23S ribosomal RNA | geneID:3345511
cg0752	2.98	0.02	putative secreted or membrane protein | putative secreted or membrane protein
cg0160	2.98	0.00	hypothetical protein cg0160 | hypothetical protein cg0160
cg1966	2.97	0.03	hypothetical protein cg1966

cg0282	2.97	0.03	hypothetical protein cg0282
cg1514	2.91	0.02	secreted protein
cg3254	2.88	0.03	hypothetical protein cg3254 | hypothetical protein cg3254
cg3218	2.87	0.00	pyruvate kinase
cg2052	2.76	0.00	putative secreted protein
cg0528	2.76	0.06	putative secreted protein
cg4005	2.71	0.01	putative secreted protein
cgtRNA_3542	2.68	0.09	
cg1291	2.65	0.01	hypothetical protein cg1291 | hypothetical protein cg1291
cg0780	2.65	0.05	membrane protein ribonuclease BN-like family
cg1513	2.48	0.17	*tnp23a*(ISCg23a), transposase-fragment | *tnp23a*(ISCg23a), transposase-fragment
cg1476	2.48	0.04	*thiC*, thiamine biosynthesis protein ThiC
cg2677	2.47	0.06	ABC-type dipeptide/oligopeptide/nickel transport system, permease component
cg2833	2.40	0.02	*cysK*, O-acetylserine (thiol)-lyase
cg2777	2.39	0.00	hypothetical protein cg2777
cg3192	2.31	0.00	putative secreted or membrane protein
cg1744	2.31	0.01	*pacL*, cation-transporting ATPase
cg3169	2.30	0.01	*pck*, phosphoenolpyruvate carboxykinase (GTP)
cg3100	2.30	0.04	*dnaK*, molecular chaperone Dnak
cg0704	2.29	0.07	hypothetical protein cg0704
cg2793	2.29	0.00	hypothetical protein cg2793
cg1884	2.25	0.01	hypothetical protein cg1884 | hypothetical protein cg1884
cg2431	2.22	0.02	putative transcriptional regulator
cg2613	2.21	0.00	*mdh*, malate dehydrogenase | mdh, malate dehydrogenase
cg3099	2.20	0.05	*grpE*, molecular chaperone GrpE (heat shock protein)
cg0683	2.16	0.05	permease
cg1229	2.15	0.01	*ykoC*, transmembrane component YkoC of energizing module of thiamin-regulated ECF transporter for hydroxymethylpyri
cg2794	2.11	0.03	hypothetical protein cg2794
cg1883	2.11	0.00	putative secreted protein
cg3008	2.11	0.01	*porA*, main cell wall channel protein
cg1917	2.10	0.06	hypothetical protein cg1917
cg1881	2.06	0.01	predicted iron-dependent peroxidase, secreted protein
cg3073	2.05	0.02	*sseA1*, probable thiosulfate sulfurtransferase protein
cg1942	2.05	0.09	putative secreted protein | putative secreted protein
cg1328	2.04	0.01	putative copper chaperone
cg2845	2.03	0.17	*pstC*, ABC-type phosphate transport system, permease component
cg3103	2.01	0.16	hypothetical protein cg3103
cg0759	0.50	0.06	*prpD2*, 2-methycitrate dehydratase
cg3047	0.50	0.02	*ackA*, acetate/propionate kinase
cg0706	0.49	0.02	conserved hypotetical membrane protein
cg3327	0.49	0.06	*dps*, starvation-induced DNA protecting protein
cg0832	0.49	0.01	*tusF*, ABC transporter, membrane spanning protein, trehalose uptake system
cg0834	0.49	0.05	*tusE*, bacterial extracellular solute-binding protein, trehalose uptake system
cg1314	0.49	0.02	*putP*, proline transport system
cg1343	0.49	0.03	*narH*, probable respiratory nitrate reductase oxidoreduct

cg3282	0.48	0.01	cation transport ATPase
cg0472	0.48	0.01	hypothetical protein cg0472 | hypothetical protein cg0472 | hypothetical protein cg0472
cg3212	0.48	0.00	putative carboxymuconolactone decarboxylase subunit
cg1052	0.47	0.00	*cmt3*, corynomycolyl transferase
cg2887	0.46	0.13	*phoS*, two component sensor kinase
cg0447	0.45	0.00	*sdhB*, succinate dehydrogenase | sdhB, succinate dehydrogenase
cg3283	0.44	0.01	hypothetical protein predicted by Glimmer
cg0046	0.44	0.09	probable ABC transport protein, ATP-binding compon
cg1603	0.43	0.01	hypothetical protein cg1603
cg3386	0.42	0.06	*tcbF*, maleylacetate reductase
cg0404	0.42	0.01	nitroreductase family
cg0798	0.41	0.25	*prpC1*, 2-methycitrate synthase
cg1090	0.41	0.07	*ggtB*, probable gamma-glutamyltranspeptidase precursor PR
cg0446	0.40	0.00	*sdhA*, succinate dehydrogenase
cg0899	0.39	0.00	*pdxT*, pyridoxine biosynthesis enzyme
cg0898	0.39	0.00	*pdxS*, pyridoxine biosynthesis enzyme
cg1434	0.39	0.12	*yggB*, small-conductance mechanosensitive channel | *yggB*, small-conductance mechanosensitive channel | *yggB*, small-conductance mechanosensitive channel
cg1108	0.38	0.01	*porC*, putative secreted protein | *porC*, putative secreted protein
cg0936	0.38	0.02	*rpf1*, resuscitation promoting factor
cg0518	0.37	0.02	*hemL*, glutamate-1-semialdehyde 2,1-aminomutase
cg1066	0.36	0.01	*urtE*, ABC-type urea uptake system, ATP binding protein
cg2953	0.36	0.02	*xylC*, benzaldehyde dehydrogenase | *xylC*, benzaldehyde dehydrogenase
cg0915	0.36	0.01	*ftsX*, putative cell division protein
cg0445	0.36	0.02	*sdhC*, succinate dehydrogenase
cg0444	0.35	0.06	*ramB*, transcriptional regulator, involved in acetate metabolism
cg3096	0.35	0.00	*ald*, alcohol degyhdrogenase
cg1341	0.35	0.01	*narI*, respiratory nitrate reductase 2 gamma chain
cg1377	0.35	0.04	*ssuC*, aliphatic sulfonates transmembrane ABC transporterprotein
cg2631	0.34	0.00	*pcaH*, protocatechuate dioxygenase beta subunit
cg1695	0.34	0.05	putative plasmid maintenance system antidote protein
cg1132	0.34	0.12	*coaA*, pantothenate kinase
cg1452	0.34	0.27	hypothetical protein cg1452
cg1376	0.34	0.01	*ssuD1*, alkanesulfonate monoxygenase
cg1380	0.33	0.01	*ssuA*, aliphatic sulfonate binding protein
cg2782	0.32	0.06	*ftn*, ferritin-like protein
cg1612	0.31	0.04	acetyltransferase | acetyltransferase
cg3107	0.31	0.01	*adhA*, Zn-dependent alcohol dehydrogenase
cg2778	0.31	0.01	hypothetical protein cg2778
cg1342	0.31	0.01	*narJ*, nitrate reductase delta chain
cg1379	0.31	0.02	*ssuB*, aliphatic sulfonates ATP-binding ABC transporterprotein
cg1344	0.30	0.00	*narG*, nitrate reductase 2, alpha subunit
cg2307	0.30	0.05	hypothetical protein cg2307
cg1737	0.29	0.01	*acn*, aconitate hydratase
cg1487	0.29	0.01	*leuC*, isopropylmalate isomerase large subunit

cg2610	0.27	0.00	ABC-type dipeptide/oligopeptide/nickel transport system, secreted component | ABC-type dipeptide/oligopeptide/nickel transport system, secreted component
cg2629	0.26	0.00	*pcaB*, ?-carboxy-cis,cis-muconate cycloisomerase | pcaB, ?-carboxy-cis,cis-muconate cycloisomerase
cg2937	0.26	0.14	ABC-type dipeptide/oligopeptide/nickel transport system, secreted component | ABC-type dipeptide/oligopeptide/nickel transport system, secreted component | ABC-type dipeptide/oligopeptide/nickel transport system, secreted component
cg1109	0.24	0.03	*porB*, anion-specific porin precursor
cg1153	0.24	0.02	*seuC*, monooxygenase for sulfonate ester utilization
cg3048	0.24	0.00	*pta*, phosphate acetyltransferase
cg0961	0.23	0.00	homoserine O-acetyltransferase
cg1156	0.22	0.02	*ssuD2*, monooxygenase for sulfonate utilization
cg1152	0.21	0.02	*seuB*, monooxygenase for sulfonate ester utilization
cg1147	0.20	0.01	*ssuI*, FMN-binding protein required for sulfonate and sulfonate ester utilization | *ssuI*, FMN-binding protein required for sulfonate and sulfonate ester utilization
cg2182	0.19	0.01	ABC-type peptide transport system, permease component
cg0228	0.19	0.07	sensor histidine kinase of two-component system, fragment
cg3195	0.18	0.00	flavin-containing monooxygenase (FMO)
cg0310	0.14	0.00	*katA*, catalase | *katA*, catalase
cg3303	0.08	0.03	transcriptional regulator PadR-like family
cg2642	0.06	0.00	*benK1*, putative benzoate transport protein | *benK1*, putative benzoate transport protein
cg2636	0.03	0.00	*catA1*, catechol 1,2-dioxygenase | *catA1*, catechol 1,2-dioxygenase

References

Baumgart, M., Luder, K., Grover, S., Gätgens, C., Besra, G.S., and Frunzke, J. (2013). IpsA, a novel LacI-type regulator, is required for inositol-derived lipid formation in *Corynebacteria* and *Mycobacteria*. BMC Biology *11*, 122.

Cremer, J., Eggeling, L., and Sahm, H. (1990). Cloning the *dapA dapB* cluster of the lysine-secreting bacterium *Corynebacterium glutamicum*. Molecular and General Genetics MGG *220*, 478-480.

Eikmanns, B.J., Thum-Schmitz, N., Eggeling, L., Lüdtke, K.-U., and Sahm, H. (1994). Nucleotide sequence, expression and transcriptional analysis of the *Corynebacterium glutamicum gltA* gene encoding citrate synthase. Microbiology *140*, 1817-1828.

Frunzke, J., Bramkamp, M., Schweitzer, J.E., and Bott, M. (2008). Population Heterogeneity in *Corynebacterium glutamicum* ATCC 13032 caused by prophage CGP3. J Bacteriol *190*, 5111-5119.

Frunzke, J., Gätgens, C., Brocker, M., and Bott, M. (2011). Control of heme homeostasis in *Corynebacterium glutamicum* by the two-component system HrrSA. Journal of Bacteriology *193*, 1212-1221.

Griffith, K.L., and Wolf, R.E., Jr. (2002). Measuring beta-galactosidase activity in bacteria: cell growth, permeabilization, and enzyme assays in 96-well arrays. Biochem Biophys Res Commun *290*, 397-402.

Hentschel, E., Mack, C., Gatgens, C., Bott, M., Brocker, M., and Frunzke, J. (2014). Phosphatase activity of the histidine kinases ensures pathway specificity of the ChrSA and HrrSA two-component systems in *Corynebacterium glutamicum*. Molecular Microbiology *92*, 1326-1342.

Heyer, A., Gatgens, C., Hentschel, E., Kalinowski, J., Bott, M., and Frunzke, J. (2012). The two-component system ChrSA is crucial for haem tolerance and interferes with HrrSA in haem-dependent gene regulation in *Corynebacterium glutamicum*. Microbiology *158*, 3020-3031.

Kinoshita, S., Udaka, S., and Shimono, M. (2004). Studies on the amino acid fermentation. Part 1. Production of L-glutamic acid by various microorganisms. The Journal of General and Applied Microbiology *50*, 331-343.

Livak, K.J., and Schmittgen, T.D. (2001). Analysis of relative gene expression data using real-time quantitative PCR and the 2(-Delta Delta C(T)) Method. Methods *25*, 402-408.

Schäfer, A., Tauch, A., Jäger, W., Kalinowski, J., Thierbach, G., and Pühler, A. (1994). Small mobilizable multi-purpose cloning vectors derived from the *Escherichia coli* plasmids pK18 and pK19: selection of defined deletions in the chromosome of *Corynebacterium glutamicum*. Gene *145*, 69-73.
